# An anaerobic bacterium host system for heterologous expression of natural product biosynthetic gene clusters

**DOI:** 10.1038/s41467-019-11673-0

**Published:** 2019-08-14

**Authors:** Tingting Hao, Zhoujie Xie, Min Wang, Liwei Liu, Yuwei Zhang, Weicang Wang, Zhao Zhang, Xuejin Zhao, Pengwei Li, Zhengyan Guo, Shushan Gao, Chunbo Lou, Guodong Zhang, Justin Merritt, Geoff P. Horsman, Yihua Chen

**Affiliations:** 1 State Key Laboratory of Microbial Resources, and CAS Key Laboratory of Microbial Physiological and Metabolic Engineering, Institute of Microbiology, Chinese Academy of Sciences, Beijing, 100101 China; 2 University of Chinese Academy of Sciences, Beijing, 100049 China; 3 MOE Key Laboratory of Industrial Fermentation Microbiology, College of Biotechnology, Tianjin University of Science & Technology, Tianjin, 300457 China; 4 Department of Food Science, University of Massachusetts, 102 Holdsworth Way, Amherst, MA 01003 USA; 5 Department of Restorative Dentistry, Oregon Health & Science University, Portland, OR 97239 USA; 6 Department of Molecular Microbiology and Immunology, Oregon Health & Science University, Portland, OR 97239 USA; 7 Department of Chemistry and Biochemistry, Wilfrid Laurier University, Waterloo, ON N2L3C5 Canada

**Keywords:** Metabolic engineering, Bacterial techniques and applications, Genetic vectors

## Abstract

Anaerobic bacteria represent an overlooked rich source of biological and chemical diversity. Due to the challenge of cultivation and genetic intractability, assessing the capability of their biosynthetic gene clusters (BGCs) for secondary metabolite production requires an efficient heterologous expression system. However, this kind of host system is still unavailable. Here, we use the facultative anaerobe *Streptococcus mutans* UA159 as a heterologous host for the expression of BGCs from anaerobic bacteria. A natural competence based large DNA fragment cloning (NabLC) technique was developed, which can move DNA fragments up to 40-kb directly and integrate a 73.7-kb BGC to the genome of *S. mutans* UA159 via three rounds of NabLC cloning. Using this system, we identify an anti-infiltration compound, mutanocyclin, from undefined BGCs from human oral bacteria. We anticipate this host system will be useful for heterologous expression of BGCs from anaerobic bacteria.

## Abstract (web summary)

Anaerobic bacteria represent a rich source of biological and chemical diversity but are difficult to cultivate and there is a lack of heterologous expression systems. Here the authors develop an expression system based on *S. mutans* UA159 for biosynthetic gene clusters from anaerobic bacteria.

## Introduction

Bacteria make numerous natural products that elicit diverse biological responses ranging from antibiotic and anticancer to immunosuppression. This pharmaceutical significance has motivated the isolation of tens of thousands of bacterial compounds, many of which have found clinical value. However, a decline in natural product discovery from traditional sources is forcing scientists to explore more microbial niches to ensure the continued supply of drugs^1–3^. For instance, aerobic microorganisms account for almost all the available drugs of microbial origin, but the natural product biosynthetic capacity of anaerobic microorganisms has been long overlooked^4,5^. Indeed, despite thriving in terrestrial habitats, marine environments, and even in human bodies, anaerobes had been considered a poor source of natural products until recently. Specifically, the rapid acquisition of genome sequence data from anaerobic bacteria has uncovered a plethora of natural product biosynthetic gene clusters (BGCs) encoding polyketides (PKs), non-ribosomal peptides (NRPs), ribosomally synthesized and post-translationally modified peptides (RiPPs), and terpenoids^5–7^. A number of compounds with specific skeletons^8,9^ or fascinating bioactivities^10–14^ have been discovered from anaerobic bacteria via traditional activity-based screening or genome mining strategies. However, natural product discovery from anaerobic bacteria remains extremely challenging due to difficulties associated with cultivation and genetic manipulation.

In addition to their pharmaceutical significance, bacterial natural products also play important physiological roles. These include functioning as defense molecules or pigments in host organisms, virulence factors of bacterial pathogens, or signals modulating the behaviors of the producers and/or their neighboring organisms including plants and mammals^15^. Significantly, the National Institutes of Health Human Microbiome Project and other studies have revealed that natural product BGCs are widely distributed in the genomes of oral and gut bacteria^6,16,17^, and the small molecules encoded by those BGCs can mediate microbiota-host interactions^17,18^ and influence human health^19–21^. Considering that the majority of the human-associated bacteria are anaerobic, appropriate methods are urgently needed to efficiently access their encoded small molecules and evaluate their clinical potential.

Because most anaerobic bacteria are unculturable, and even culturable species may not have established genetic systems, heterologous expression of their predicted BGCs can be used to circumvent both problems. Previous studies revealed that BGCs were more successfully expressed in heterologous hosts that were more closely related to the native producer^22,23^. This implies that anaerobic model microorganisms should be phylogenetically adjacent to anaerobes harboring target BGCs because they likely share similar growth conditions, G + C content, and regulatory systems. Unfortunately, current heterologous hosts are limited to expressing natural product BGCs from aerobic microbes, and popular heterologous expression hosts like *Streptomyces*, Myxobacteria, and *Bacillus subtilis* are obligate aerobes, with the exception of the Gram-negative facultative anaerobe *E. coli*^22,23^. In practice however, slow growth rates under anaerobic conditions restrict even *E. coli* to aerobic conditions for heterologous expression of natural product BGCs.

An ideal anaerobic host for heterologous expression of natural product BGCs should (i*)* be safe to manipulate in the laboratory; (ii) grow rapidly under anaerobic conditions; (iii) have clear genetic and metabolic backgrounds; (iv) have versatile genetic tools and readily accept large DNA fragments to accommodate the 10–120 kb size range of most natural product BGCs; and (v) possess the requisite precursor molecules to support the biosynthesis of diverse natural products. Genomic analysis of anaerobic bacteria from various environments, including the human body, revealed high average BGC abundances from some families within the predominantly Gram-positive Clostridia and Bacilli classes of the Firmicutes phylum^5,6^. Clostridia members are obligate anaerobes infamous for being difficult to culture, which makes them unattractive as heterologous expression hosts. The Bacilli are distinguished from the Clostridia by their capacity for aerobic respiration, and many Bacilli strains are aerotolerant or facultative anaerobes that can grow well without fastidious cultivation. In addition, among the top 30 families with high average BGC abundances in the human microbiome, four families are from the Bacilli class, with three (Aerococcaceae, Carnobacteriaceae, and Streptococcaceae) belonging to the Lactobacillales order^6^. We therefore focused our analysis on these three families from which to choose an appropriate host for BGCs heterologous expression. The well-established protocols and genetic tools available for *Streptococcus* directed our attention to this genus^24^.

*Streptococcus* is a well-studied genus of Gram-positive bacteria in the Streptococcaceae family. Certain species of *Streptococcus* are pathogenic and may cause infectious diseases like bacterial pneumonia, meningitis, and endocarditis. However, several species used in producing yogurt or cheese, and many more are important components of the human microbiota. We previously analyzed the genomes of 10,038 *Streptococcus* strains and identified the facultative anaerobe *Streptococcus mutans* (*S. mutans*) as a rich source of PKs, NRPs, hybrid PK-NRPs, and RiPPs^25^. *S. mutans*, which is proposed to be a contributor to tooth decay, is naturally present in the human oral microbiota. The prototype strain of this species, *Streptococcus mutans* UA159, is safe and easy for genetic manipulation under laboratory conditions. It prefers to grow in anaerobic conditions with a relatively short doubling time (40–60 min), but is moderately aerotolerant as well. Complete sequencing and annotation of the *S. mutans* UA159 genome indicated a capacity to supply common precursors for the biosynthesis of PKs, NRPs, RiPPs, and terpenoids^26^. A series of hybrid PK-NRPs (mutanobactins) and two RiPPs (mutacin IV and V) were discovered in *S. mutans* UA159, revealing its potential as a producer of diverse natural products^13,14,27^. The existence of expedient genetic tools for introducing large DNA fragments into *S. mutans* UA159 would make this strain an attractive host for heterologous expression of BGCs from anaerobes belonging to the Streptococcaceae family, with potential utility in the Bacilli class or more broadly to other Firmicutes.

Herein, we describe the development of a natural competence based large DNA fragment cloning (NabLC) technique to mobilize target genomic DNA fragments up to 40-kb for direct insertion into a desired genomic locus of the acceptor strain *S. mutans* UA159. Several BGCs from different anaerobic bacterial strains, as large as 73.7-kb, are successfully cloned into the *S. mutans* UA159 genome to demonstrate the reliability of the NabLC technique for large DNA fragment cloning. We subsequently validate *S. mutans* UA159 as a host for anaerobic BGCs by functionally expressing a known pyrazinone BGC from *Staphylococcus epidermidis* (*Sa. epidermidis*) ATCC 35984 and two unidentified BGCs, BGC1 and BGC4, from human oral bacteria *S. mutans* 35 and *S. mutans* NMT4863 respectively. The activation of BGC4 leads to the discovery of a (2*E*)-decenoyl dipeptide SNC1–465. The product of BGC1 is identified as mutanocyclin, a tetramic acid with anti-infiltration activity. Because BGC1 is only found in the genomes of *Streptococcus* strains living in human and primate mouths, mutanocyclin may represent an adaptive response to this oral environment.

## Results

### Development of the NabLC technique in *S. mutans* UA159

**Fig. 1:**
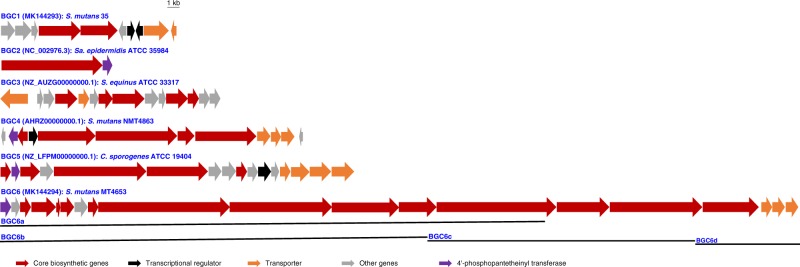
BGCs cloned in this study. The BGCs cloned in this study were from different anaerobic bacteria, including BGC1, BGC3, BGC4, BGC6 from *Streptococcus* strains; BGC2 from a *Staphylococcus* strain and BGC5 from a *Clostridium* strain

We firstly tried to construct a vector-based heterologous expression system in *S. mutans* UA159 using the Cas9-Assisted Targeting of Chromosome (CATCH) segments technique, which has successfully cloned large DNA fragments from *Streptomyces* and *E. coli*^28^. However, CATCH cloning of four BGCs—the 38.6-kb mutanobactin gene cluster from *S. mutans* UA159, and BGC1, BGC4, BGC6 from other *S. mutans* strains (Fig. 1)—yielded positive transformants only for BGC1 from *S. mutans* 35, which at 13.1-kb is the shortest among the four. The difficulties in cloning large DNA fragments from *Streptococcus* with the CATCH system forced us to consider other DNA cloning techniques.

**Fig. 2:**
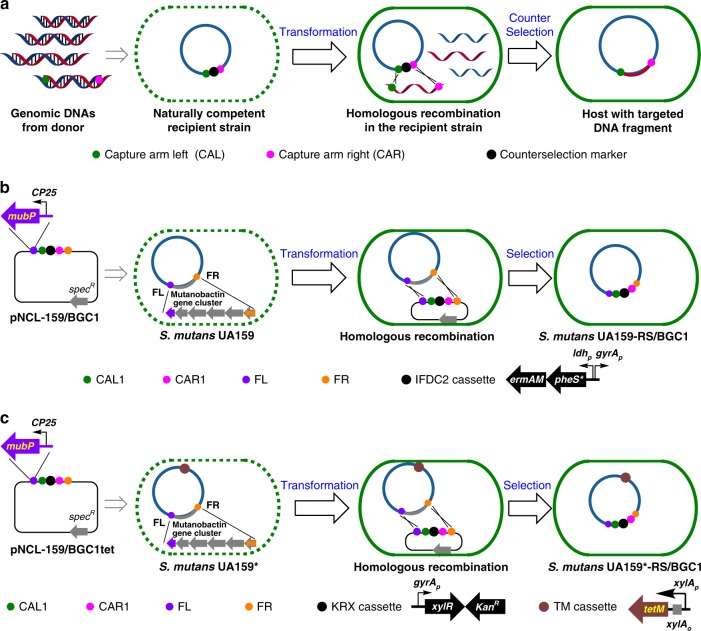
Development of the NabLC technique in *S. mutans* UA159. **a** Description of the NabLC technique. The whole genomic DNAs from donor were transformed to the *S. mutans* UA159-derived recipient strain via natural competence and internalized as ssDNAs. The target DNA fragment was inserted into the genome of *S. mutans* UA159 by homologous recombination and the recombinant strains were screened using the counterselection marker. **b** Construction of the recipient strain *S. mutans* UA159-RS/BGC1 using the PheS^*^ based counterselection system. FL, CAL1, IFDC2 cassette, CAR1, FR were ligated to pFW5 to generate pNCL-159/BGC1 and then transformed to *S. mutans* UA159 in replacement of the mutanobactin gene cluster to obtain the recipient strain *S. mutans* UA159-RS/BGC1. **c** Construction of the recipient strain *S. mutans* UA159^*^-RS/BGC1 using the *tetM* based counterselection system. The TM cassette was first integrated into the genome of *S. mutans* UA159, and the IFDC2 cassette was replaced by the KRX cassette. *kan*^*R*^, kanamycin resistance; *spec*^*R*^, spectinomycin resistance

It has been well established that many *Streptococcus* strains, including *S. mutans* UA159, can actively internalize exogenous DNA from the environment via natural competence^29^. In *S. mutans*, natural competence is tightly regulated by a network of genes under the control of at least two peptide signaling molecules, namely, the competence-stimulating peptide (CSP) and the *comX*-inducing peptide (XIP). These peptides can be added to early-exponential-phase cultures of *S. mutans* to enhance the expression of *comX*, an alternative sigma factor responsible for transcribing the late competence effector genes encoding the DNA uptake apparatus^30^. Previous reports on natural transformation of large DNAs by *B. subtilis* and *Ralstonia solanacearum*^31–33^ inspired our pursuit of a natural competence-based approach in *S. mutans* UA159. Our strategy for natural competence-based large DNA fragment cloning (NabLC) is illustrated in Fig. 2a. First, an *S. mutans* UA159-derived recipient strain is constructed by cloning a capture cassette into a desired locus of *S. mutans* UA159 genome. The capture cassette contains a counterselection marker flanked by left and right capture arms (CAL and CAR, respectively), each of which is a DNA fragment (about 2-kb) matching the left or right end of the target gene cluster. The recombinant strains are then cultured under selective pressure such that survival requires replacing the counterselection marker with the target gene cluster via homologous recombination.

As proof of concept, the 13.1-kb BGC1 from *S. mutans* 35 was cloned using the NabLC technique. We endeavored to incorporate BGC1 into the genome of *S. mutans* UA159 by replacing the mutanobactin gene cluster (Fig. 2b). To construct the *S. mutans* UA159 recipient strain, the 0.8-kb FL and 0.9-kb FR fragments flanking the mutanobactin gene cluster were cloned and inserted into the suicide vector pFW5 to generate pNCL-159. The FL fragment included a constitutive promoter CP25 in front of the *mubP* gene^34^ encoding a Sfp-type phosphopantetheinyl transferase, which will facilitate the functional expression of polyketide synthases (PKSs) and non-ribosomal peptide synthetases (NRPSs) by post-translationally adding the phosphopantetheinyl arms to their thiolation domains^35^. The capture cassette for BGC1 was constructed using IFDC2, a well-established counterselection system in *S. mutans*. IFDC2 contains the erythromycin resistance gene (*ermAM*) and the gene encoding PheS^*^, which is an A294G mutant of the *α*-subunit of *L*-phenylalanine-tRNA synthetase that can kill cells by incorporating *p*-chloro-phenylalanine (*p*-Cl-Phe) into proteins^36^. A 2.0-kb capture arm from BGC1’s left end (CAL1), a 1.8-kb capture arm from its right end (CAR1) and the 2.1-kb IFDC2 cassette were cloned and inserted into pNCL-159 to generate pNCL-159/BGC1, in which the five inserted elements were aligned as FL-CAL1-IFDC2-CAR1-FR. The recipient strain *S. mutans* UA159-RS/BGC1, in which the mutanobactin gene cluster was replaced by the capture cassette CAR1-IFDC2-CAL1, was obtained by transforming pNCL-159/BGC1 into *S. mutans* UA159 and screening for transformants resistant to erythromycin and sensitive to spectinomycin. *S. mutans* UA159-RS/BGC1 was then transformed with *S. mutans* 35 genomic DNAs via natural transformation, and colonies that could survive on plates containing 4 mg/mL *p*-Cl-Phe were picked as positive transformants. Since a high false positive rate was observed (Supplementary Tables 1 and 2), the strains harboring BGC1 were further tested for sensitivity to erythromycin and PCR amplification (Supplementary Fig. 1). The correct *S. mutans* UA159 strains possessing a complete BGC1 cluster were named *S. mutans* UA159/BGC1.

### Optimization of the NabLC technique in *S. mutans* UA159

The screening process revealed that the high rate of false positives arising from the PheS^*^-based counterselection system significantly decreased the screening efficiency. To reduce the false positive rate, we constructed a counterselection system based on the tetracycline resistance gene (*tetM*) as illustrated in Fig. 2c. First, a tetracycline resistance gene cassette (TM cassette), in which *tetM* is downstream of the *xylA*_*p*_ promoter and the XylR repressor binding site *xylA*_*o*_, was integrated into the genome of *S. mutans* UA159 by replacing the *smu2080-2081* operon to afford *S. mutans* UA159^*^. Previous work showed that deleting this operon had no obvious toxicity to *S. mutans* UA159^37^. The repressor-encoding gene *xylR* was then used as a counterselection marker in *S. mutans* UA159^*^. Specifically, *xylR* was put under the control of the constitutive *gyrA*_*p*_ promoter and combined with a kanamycin resistance gene to generate the KRX cassette, which replaced the IFDC2 cassette in pNCL-159/BGC1 to generate pNCL-159/BGC1tet. The corresponding recipient strain *S. mutans* UA159^*^-RS/BGC1 was obtained by transforming pNCL-159/BGC1tet into *S. mutans* UA159^*^ and screening for transformants resistant to kanamycin and sensitive to spectinomycin. When *S. mutans* UA159^*^-RS/BGC1 was cultured on plates with 15 μg/mL tetracycline, the background survival rate was determined to be (1.0 ± 0.1) × 10^−8^ (mean ± standard deviation, *n* = 3; same for the followings except specifying), which was about one order of magnitude lower than *S. mutans* UA159-RS/BGC1 on plates with 4 mg/mL *p*-Cl-Phe ((2.0 ± 0.8) × 10^–7^) (Table S1), suggesting superior screening efficiency of the *tetM*-based counterselection marker.

The higher screening efficiency of the *tetM*-based counterselection system was verified by transforming both recipient strains, *S. mutans* UA159-RS/BGC1 and UA159^*^-RS/BGC1, with the same amount of *S. mutans* 35 genomic DNA. Sixty-four colonies that could survive on plates with *p*-Cl-Phe or tetracycline were picked randomly in each case, and recombinant strains harboring BGC1 were then screened for their sensitivities to erythromycin or kanamycin. Verification by colony PCR (Supplementary Fig. 1) revealed that the hit rate for BGC1 incorporation was about one order of magnitude higher for *S. mutans* UA159^*^/BGC1 than for *S. mutans* UA159/BGC1 (Supplementary Table 2). We therefore chose to use the *tetM*-based counterselection system for BGC cloning in *S. mutans* UA159^*^.

### Cloning BGCs with the NabLC technique in *S. mutans* UA159^*^

Encouraged by the successful incorporation of BGC1 into the *S. mutans* UA159^*^ genome, we used the NabLC technique to clone different BGCs from diverse anaerobic bacteria including *Streptococcus*, *Staphylococcus*, and *Clostridium*. The seven BGCs (BGC2-6 and BGC6a-6b) ranging from 7.9- to 73.7-kb (Fig. 1) were tested using the *tetM* based counterselection system. For DNA fragments smaller than 40 kb, correct recombinant strains could be identified by PCR screening 64 positive transformants on tetracycline plates, regardless of their bacterial sources (Supplementary Fig. 1). However, recombinant *S. mutans* UA159^*^ strains were not identified from 128 colonies screened for each of BGC6a (50.6-kb) and BGC6 (73.7-kb), thus setting an upper size limit of the NabLC technique in *S. mutans* UA159^*^. We also note a surprisingly low positive hit rate for the small 7.9-kb BGC2 from *Staphylococcus epidermidis* ATCC 35984, implying that the bacterial source of DNAs can affect cloning efficiency (Table 1).

**Table 1:** BGCs cloned with the NabLC technique in *S. mutans* UA159^*^

BGCs	Types	Sizes (kb)	Strains	Correct /picked
BGC1	NRPS/PKS	13.1	*S. mutans* 35	15/64
BGC2	NRPS	7.9	*Sa. epidermidis* ATCC 35984	1/64
BGC3	RiPP	16.5	*S. equinus* ATCC 33317	26/32
BGC4	NRPS/PKS	21.9	*S. mutans* NMT4863	2/64
BGC5	NRPS	28.1	*C. sporogenes* ATCC 19404	5/48
BGC6	PKS	73.7	*S. mutans* MT4653	0/128
BGC6a	PKS	50.6	*S. mutans* MT4653	0/128
BGC6b	PKS	40.0	*S. mutans* MT4653	1/64
BGC6c	PKS	23.6	*S. mutans* MT4653	2/64
BGC6d	PKS	10.1	*S. mutans* MT4653	58/64

Elimination of the counterselection cassette (e.g., KRX cassette) represents a useful feature of the NabLC strategy that enables iterative rounds of assembly to stitch together large BGCs. For example, although the entire 73.7-kb BGC6 fragment was too large to clone in one time, it served an opportunity to test multiple rounds of assembly to obtain large BGCs. And, iterative rounds of NabLC cloning were used to reassemble the complete cluster from three fragments BGC6b-d (Supplementary Fig. 2). With *S. mutans* UA159^*^/BGC6b strain harboring the 40-kb fragment in hand (Round I), the remaining 33.7 kb of BGC6 was added by sequentially cloning the 23.6-kb BGC6c (Round II) and the 10.1-kb BGC6d (Round III). In summary, exploiting KRX cassette elimination at each cloning step enabled iterative assembly of the complete 73.7-kb BGC6 in *S. mutans* UA159^*^ in three cloning steps.

### Activation of BGC2 in *S. mutans* UA159^*^

**Fig. 3:**
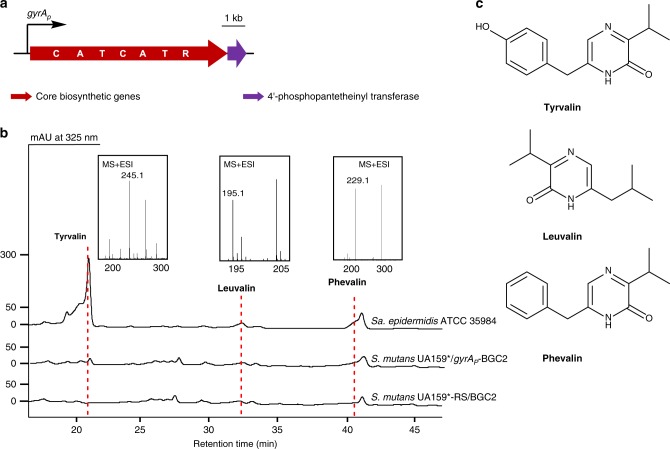
Activation of BGC2 and HPLC profiles of *S. mutans* UA159^*^/*gyrA*_*P*_-BGC2. **a** A constitutive promoter *gyrA*_*p*_ was inserted upstream of BGC2 in *S. mutans* UA159^*^/BGC2 to obtain *S. mutans* UA159^*^/*gyrA*_*P*_-BGC2. The functional NRPS domains are indicated in bold: A, adenylation domain; C, condensation domain; R, terminal reductase; T, thiolation domain. **b** HPLC traces of the supernatant extracts of *S. mutans* UA159^*^/*gyrA*_*P*_-BGC2, *Sa. epidermidis* ATCC 35984 (a positive control) and *S. mutans* UA159^*^-RS/BGC2 (a negative control). The LC-MS extracted ion count chromatograms of three pyrazinone compounds are also shown. **c** Structures of the three pyrazinone compounds

After successfully capturing different BGCs on the *S. mutans* UA159^*^ genome using the NabLC technique, we set out to test the utility of *S. mutans* UA159^*^ as a host for functionally expressing BGCs as judged by detection of their encoded natural products. Among the six captured clusters, only the products encoded by BGC2 have been characterized. Several pathogenic *Staphylococcus* strains, including *Sa. epidermidis*, *Sa. aureus*, *Sa. capitis*, and *Sa. lugdenensis* harbor BGC2, which encodes an NRPS responsible for the biosynthesis of the pyrazinones tyrvalin, phevalin, and leuvalin^38^. To activate BGC2, we incorporated the gyrase constitutive promoter *gyrA*_*P*_ upstream of BGC2 in *S. mutans* UA159^*^/BGC2 to obtain *S. mutans* UA159^*^/*gyrA*_*P*_-BGC2. The three pyrazinones were detected by HPLC and LC-MS analysis of extracts of *S. mutans* UA159^*^/*gyrA*_*P*_-BGC2, but not from extracts of the negative control *S. mutans* UA159^*^-RS/BGC2 (Fig. 3 and Supplementary Fig. 3). These results clearly demonstrated the utility of *S. mutans* UA159^*^ as a heterologous host for functional expression of NRPS gene clusters.

### Distribution of BGC1

Of the five remaining BGCs, all of which encode unknown products, BGC1 stands out for being confined to *Streptococcus* species found in the mouths of primates, including humans. Database searching showed that gene clusters having the same organization as BGC1 were found in 25 *S. mutans* and two other *Streptococcus* strains, *S. troglodytae* TKU31^39^, and *S. macacae* NCTC 11558^40^ (Supplementary Fig. 4). *S. troglodytae* TKU31 was isolated from the chimpanzee oral cavity, and *S. macacae* NCTC 11558 was isolated from the dental plaque of a monkey. We firstly attempted to identify the product of BGC1 in native producers by PCR-screening the collected *S. mutans* isolates. This process identified three more strains (B30, B409, and B608), in addition to *S. mutans* 35, that contain BGC1. Unfortunately, all four strains were recalcitrant to genetic manipulation in our hands, impeding the investigations of BGC1 products in those native strains.

### Activation of BGC1 in *S. mutans* UA159^*^

**Fig. 4:**
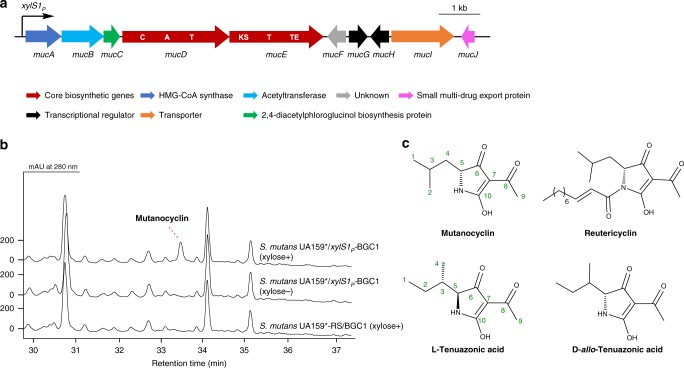
Activation of BGC1 and metabolic profiles of *S. mutans* UA159^*^/*xylS1*_*P*_-BGC1. **a** The xylose-inducible promoter *xylS1*_*P*_ was inserted upstream of BGC1 in *S. mutans* UA159^*^/BGC1 to obtain *S. mutans* UA159^*^/*xylS1*_*P*_-BGC1. Functional NRPS and PKS domains are indicated in bold: A, adenylation domain; C, condensation domain; KS, ketosynthase; T, thiolation domain; TE, thioesterase. **b** HPLC traces of the supernatant extracts of *S. mutans* UA159^*^/*xylS1*_*P*_-BGC1 induced with xylose, *S. mutans* UA159^*^/*xylS1*_*P*_-BGC1 without induction and *S. mutans* UA159^*^-RS/BGC1 induced with xylose were used as negative controls. **c** Structures of mutanocyclin and its congeners

To activate BGC1 in the *S. mutans* UA159^*^ host, the xylose-inducible promoter *xylS1*_*P*_ was inserted upstream of the gene cluster in *S. mutans* UA159^*^/BGC1 to generate *S. mutans* UA159^*^/*xylS1*_*P*_-BGC1. When *S. mutans* UA159^*^/*xylS1*_*P*_-BGC1 was cultured in ASS (Artificial Saliva Substitutes) medium, a peak with a retention time of 33.4 min was observed only in the xylose induced culture (Fig. 4), suggesting that it is the product of BGC1.

Due to the similarity between BGC1 and the reutericyclin gene clusters from *Lactobacillus reuteri* strains^41^ (Supplementary Fig. 4), we previously proposed that BGC1 encodes a reutericyclin-like compound^25^. Indeed, the chemical formula of the BGC1 product was C_10_H_15_NO_3_ (HR-MS, *m*/*z* [M + H]^+^ 198.1124, calculated 198.1125), consistent with a tetramic acid reutericyclin core lacking the (*E*)-dec-2-enoic acyl chain. This was supported by ^1^H NMR spectra that were almost identical to ^1^H NMR data reported for the chemically synthesized tetramic acid core of reutericyclin^42^ (Supplementary Table 3, Supplementary Fig. 5). Optical rotation analysis confirmed that the product of BGC1 has an (*R*)-configuration at C-5 as reutericyclin. Final structural assignment was accomplished by chemical synthesis of the proposed product from *Boc*-protected *D*-Leu starting material (Supplementary Fig. 6), which revealed chemical shifts and coupling constants matching the isolated compound and a single HPLC peak arising from co-injection (Supplementary Tables 4 and 5, Supplementary Fig. 7a). The structurally confirmed BGC1 product was named mutanocyclin (Fig. 4c).

To verify mutanocyclin as the bona fide natural product of BGC1 in *S. mutans* wild-type strains, it was detected by HPLC and HR-LC-MS from cultures of *S. mutans* 35, B30, B409, and B608 grown in ASS medium (Supplementary Fig. 10). In addition, our inability to detect reutericyclin (Supplementary Fig. 10) further suggests mutanocyclin as the true product of BGC1 in *S. mutans* isolates.

### Bioactivities of mutanocyclin

Various mutanocyclin congeners have been evaluated for biological activity. The *N*-fatty acyl congener reutericyclin displays good inhibition activities against Gram-positive bacteria^43^ while the mycotoxin *L*-tenuazonic acid^44^ possesses antibacterial, antitumor, antiviral and phytotoxic activities (Fig. 4c). Although mutanocyclin was not discovered as a natural product before, it was chemically synthesized as racemate and found to have no considerable cytotoxicity^45^. Consistent with previous work^46^, we detected no significant antibacterial activity of mutanocyclin against *Escherichia coli*, *B. subtilis*, and the oral commensal bacteria *Streptococcus oralis*, *Streptococcus sanguinis*, *Streptococcus gordonii*, and *Veillonella atypica* (Supplementary Table 7).

The association of BGC1 to organisms specific to primate oral environment inspired us to evaluate a possible immunomodulatory activity of mutanocyclin using a Matrigel plug assay in C57BL/6 mice. We treated mice with subcutaneous injection of 0.25 mL Matrigel containing mutanocyclin (10 μg) or vehicle, and then quantified immune cell infiltration into the implanted Matrigel plug. Flow cytometry analysis showed that mutanocyclin significantly suppressed infiltration of leukocytes (CD45^+^ cells) into the Matrigel plug, suggesting an anti-inflammatory effect of mutanocyclin in vivo (Supplementary Fig. 11).

### Activation of BGC4 in *S. mutans* UA159^*^

**Fig. 5:**
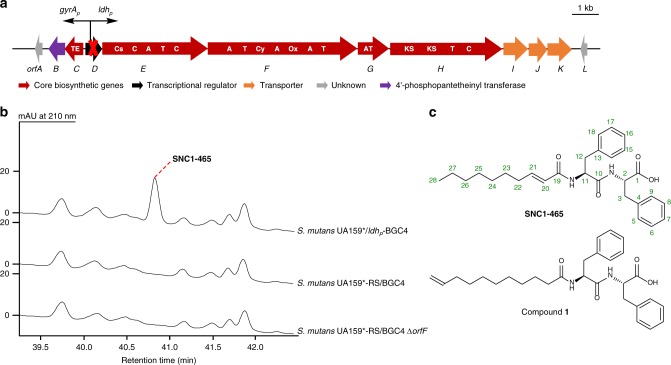
Activation of BGC4 and metabolic profiles of *S. mutans* UA159^*^/*ldh*_*P*_-BGC4. **a** Two constitutive promoters *gyrA*_*p*_ and *ldh*_*P*_ were inserted to replace the transcriptional regulatory gene *orfD* in *S. mutans* UA159^*^/BGC4 to obtain *S. mutans* UA159^*^/*ldh*_*P*_-BGC4. Functional NRPS and PKS domains are indicated in bold: A, adenylation domain; C, condensation domain; Cs, starter condensation domain; Cy, heterocyclization domain; Ox, oxidative domain; AT, acyl-transferase; KS, ketosynthase; T, thiolation domain; TE, thioesterase. **b** HPLC traces of the supernatant extracts of *S. mutans* UA159^*^/*ldh*_*P*_-BGC4, *S. mutans* UA159^*^-RS/BGC4 and *S. mutans* UA159^*^/*ldh*_*P*_-BGC4 ∆*orfF* were used as negative controls. **c** Structures of SNC1-465 and its analogue compound **1**

Complete BGC4 has been found in 4 *S. mutans* strains and *Streptococcus mitis* SK597 (accession number: NZ_AEDV01000073.1) from human mouth and *Streptococcus equinus* Sb10 (accession number: NZ_FNJY01000001.1) from horse feces. To activate BGC4, we constructed *S. mutans* strain UA159^*^/*ldh*_*P*_-BGC4 by insertion of two oppositely oriented constitutive promoters *gyrA*_*p*_ and *ldh*_*p*_ to replace *orfD*, a putative TetR/AcrR family regulatory gene (Fig. 5). When *S. mutans* UA159^*^/*ldh*_*P*_-BGC4 was cultured in ASS medium (pH 7.0), a peak with a retention time of 40.8 min that was absent in the negative control *S. mutans* UA159^*^-RS/BGC4 was noticed (Fig. 5). Subsequently, we knocked out one of the structure gene *orfF* to generate *S. mutans* UA159^*^/*ldh*_*P*_-BGC4 ∆*orfF*, in which the 40.8 min peak could not be detected, confirming it was produced by BGC4. High resolution-mass spectrum analysis of the compound revealed its chemical formula as C_28_H_36_N_2_O_4_ (HR-MS, *m*/*z* [M + H]^+^ 465.2761, calculated 465.2753) (Supplementary Fig. 12), and it was named as SNC1-465. Further NMR analysis assigned SNC1-465 as a (2*E*)-decenoyl dipeptide containing two phenylalanine residues (Supplementary Table 6, Supplementary Figs. 12, 13 and 14), which were both assigned as L-configuration by the Marfey’s method^47^ (Fig. 5c, Supplementary Fig. 15).

SNC1-465 exhibited no significant antibacterial activity against *E. coli*, *B. subtilis*, *Sa. aureus* and the oral commensal bacteria *S. oralis*, *S. sanguinis*, *S. gordonii*, and *S. mitis*. It exhibited no inhibition activity against *Candida albicans*, either. A 10-undecenoyl di-phenylalanine analogue of SNC1-465 (compound **1** in Fig. 5c) was reported to have whitening efficacy by inhibiting tyrosinase, the rate-limiting enzyme in melanin biosynthesis^48^. We tested the inhibitory activity of SNC1-465 on mushroom tyrosinase. Unfortunately, no significant inhibition on tyrosinase activity was detected for SNC1-465 (Supplementary Table 8).

## Discussion

The secondary metabolite biosynthetic capacity of anaerobic bacteria has been long overlooked, but the post genomic era is reinvigorating interest in probing natural product chemical space and understanding fitness mechanisms of anaerobic bacteria in unusual habitats^17,18^. However, despite the plethora of BGCs observed in anaerobic bacteria genomes, accessing their encoded products is usually complicated by the recalcitrance of these organisms to both laboratory cultivation and genetic manipulation. We therefore selected *S. mutans* UA159 as a heterologous expression host for BGC mining because: (i) it is a well-studied model strain exhibiting rapid growth under anaerobic conditions, (ii) genetic tools are well-established, and (iii) it possesses the capacity to synthesize versatile natural products^26^. Unfortunately, we could not clone large DNA fragments from *S. mutans* using the established CATCH technique, which yielded successful genomic integration of only the 13.1-kb BGC1. This result implied that the CATCH vector/*E. coli* (transit strain) system may not be suitable for cloning large DNA fragments from Gram-positive anaerobic *S. mutans* with high A + T content (about 63%).

To overcome this barrier, we exploited the natural competence of *S. mutans* UA159 to develop the NabLC approach for incorporating large target DNA fragments directly into a desired genomic locus of the receptor strain. The NabLC technique mobilized DNA fragments up to 40 kb for direct incorporation into the genome of *S. mutans* UA159^*^. BGCs from varied Gram-positive anaerobic bacteria such as *Staphylococcus* and *Clostridium* were also cloned successfully. In addition, the 73.7-kb BGC6 was pieced together on the genome of *S. mutans* UA159^*^ by sequential assembly with the NabLC technique, revealing its utility for cloning large DNA fragments. Although many currently available vector-based techniques for cloning and reconstruction of large BGCs^28,49^ have the advantages of easy genetic manipulations and transferability among different hosts, these approaches often suffer from vector instability and incompatibility. In contrast to vector-based approaches, cloning DNA fragments directly into host genomes^31–33^ may serve as valuable alternatives, and the NabLC technique we have developed may be especially useful for bacteria with natural competence.

The NabLC technique establishes a robust system for heterologously expressing natural product BGCs from anaerobic bacteria, and is particularly suited to expressing BGCs from bacteria of the Bacilli class, to which our chosen host *S. mutans* UA159^*^ belongs. Using the NabLC technique, we cloned the previously characterized BGC2 from *Sa. epidermidis* ATCC 35984, which belongs to the Staphylococcaceae family in the order of Bacillales. Using the *gyrA*_*P*_ constitutive promoter, BGC2 was successfully activated in *S. mutans* UA159^*^ to yield its three pyrazinone products, thus demonstrating the feasibility of this system for natural product discovery from anaerobic bacteria. We also used NabLC to clone and heterologously activate BGC1 from *S. mutans* that were recalcitrant to genetic manipulation, and this produced the tetramic acid compound mutanocyclin. Importantly, we engineered the *S. mutans* UA159^*^ host to constitutively express the phosphopantetheinyl transferase MubP to facilitate the functional expression of BGCs encoded PKS and NRPS pathways. In the case of BGC1, which contains *mucD* and *mucE* that encode an NRPS and a PKS protein respectively, their thiolation domains should be activated to holo-form by MubP. In addition, MucE is a trans-AT PKS requiring a standalone acyltransferase to load malonyl-CoA chain extender units onto its thiolation domain. The absence of acyltransferase-encoding genes in BGC1 indicates that proficient acyltransferase(s) are encoded by the *S. mutans* UA159^*^ host genome, suggesting this host strain is well-suited for expressing trans-AT PKSs. Finally, attempts to activate the remaining four BGCs cloned in this study have resulted in the detection of the BGC4-encoded compound SNC1-465, which further highlights the BGC mining potential of the NabLC*/S. mutans* UA159^*^ heterologous expression system.

Although previously chemically synthesized as a congener of reutericyclin^42^, we herein report the discovery of mutanocyclin as a natural product. The mutanocyclin-encoding BGC1 is very similar to the BGC for reutericyclin, which exists exclusively in *Lactobacillus* strains from sourdough, fermented soybean, and pig feed (Supplementary Fig. 4). In contrast, BGC1 is found only in *Streptococcus* strains inhabiting the mouths of human and other primates. As a low molecular weight antibiotic isolated from *Lactobacillus* genus, reutericyclin shows broad-spectrum bacteriostatic and bactericidal activity against Gram-positive bacteria, including *Staphylococcus aureus*, *Enterococcus faecium*, and *Clostridium difficile*^41^. Mutanocyclin, the deacylated version of reutericyclin, lacks antibiotic activity and instead inhibits leukocyte chemotaxis in a murine Matrigel assay. The contrasting structure-bioactivity profiles of reutericyclin and mutanocyclin may reflect distinct selection pressures in their respective environments: Lactobaccili in nutrient-rich food stuffs mobilize reutericyclin as a weapon against competing microbes in the fertile environments; whereas mutanocyclin may play an immunomodulatory role as part of microbiome-host interactions. Database searching revealed two more BGCs very similar to both BGC1 and the reutericyclin gene cluster: one in the genome of *Streptococcus orisasini* SH06 isolated from a healthy thoroughbred gastrointestinal tract^50^; and another in *Streptococcus* sp. HMSC068F04 isolated from human sputum (Supplementary Fig. 4). Although further studies are needed to identify products encoded by these two BGCs, the producer organism habitats suggest similar activities as mutanocyclin, especially for the latter.

*L*-Tenuazonic acid, another natural product analog of mutanocyclin, was discovered as a phytotoxin from fungi strains *Alternaria*, *Aspergillus*, *Pyricularia*, and *Ulocladium*^51^. Its C5-epimer *D*-*allo*-tenuazonic acid differs from mutanocyclin only in the position of the methyl branch. Tenuazonic acid has been shown to be biosynthesized by a hybrid NRPS-PKS protein TAS1. Its C7-acetyl group is derived from acetoacetyl-CoA incorporated at the first biosynthetic step, and its pyrrolidine ring is formed via Dieckmann condensation catalyzed by an atypical ketosynthase domain^44^ (Supplementary Fig. 16a). The proposed biosynthetic pathway of reutericyclin^39^ involves addition of the C7-acetyl group as the final step, with thioesterase-catalyzed lactam bond formation to afford the pyrrolidine ring (Supplementary Fig. 16b). Overall, the high similarity among BGC1 and the BGC of reutericyclin suggests an analogous biosynthetic logic operating for mutanocyclin. The main difference between reutericyclin and mutanocyclin is the *N*-fatty acyl chain decoration. With the presence of a starter condensation domain in MutD similar to RtcN^41^, we speculated that the *N*-fatty acyl chain was initially part of mutanocyclin and later removed during its biosynthesis. This hypothesis is currently under investigation.

Activation of BGC4 in *S. mutans* UA159^*^ resulted in the lipopeptide compound SNC1-465. From our analysis of BGC4, SNC1-465 is likely to be a side product, since an *L*-cysteine is predicted to be incorporated into SNC1-465 by *orfF* to form a thiazole ring as described in epothilone^52^ and myxothiazol^53^ biosynthesis (Supplementary Fig. 17a). This compound may then serve as a substrate for the following PKS encoded by *orfH*. Production of SNC1-465 by BGC4 is reminiscent of the production of *N*-myristoyl-*D*-Asn by the colibactin gene cluster in *E. coli*, in which *N*-myristoyl-*D*-Asn is cleaved from the final product (Supplementary Fig. 17b)^54^. Also, it could not be excluded that SNC1-465 is a premature product detached from the NRPS assembly line of BGC4.

In conclusion, we have established *S. mutans* UA159 as a heterologous host for expressing BGCs from anaerobic bacteria that have been identified by recent genome mining efforts. In addition, we developed the NabLC technique to facilitate the capture of large DNA fragments in the genome of *S. mutans* UA159. Successful production of the pyrazinone compounds (BGC2) and mutanocyclin (BGC1) revealed the utility of this system for functionally expressing BGCs from different anaerobic bacteria. Anaerobic heterologous expression systems like the one developed in this study will help accommodate characterization of the exponentially-increasing number of BGCs identified from anaerobic bacterial genomic data. We anticipate this system will facilitate discovery of bioactive natural products from anaerobic sources and accelerate the elucidation of molecular fitness mechanisms in anaerobes.

## Methods

### Bacterial strains and culture conditions

Bacterial strains and plasmids used in this study are listed in Supplementary Data 1. For preparation of genomic DNAs, all *Streptococcus* strains and *Staphylococcus* strains were grown in brain heart infusion broth (BHI; OXOID LTD., Basingstoke, England) or on BHI agar plates; *Clostridium* strains were grown in reinforced medium for *Clostridia* (Hopebio Co., Qingdao, China). For transformation experiments, cells were maintained in Todd-Hewitt medium (Becton-Dickinson, Sparks, MD) supplemented with 0.3% yeast extract (THYE). The chemically defined medium ASS (Artificial Saliva Substitutes) was modified from the published ASS medium^55^ by adding extra ingredients: *L*-Val (0.12 g/L), *L*-Phe (0.17 g/L), *L*-Asp (0.13 g/L), *L*-Asn (0.13 g/L), *L*-Ala (0.09 g/L), and peptone (0.1%). For the selection of antibiotic-resistant colonies, BHI plates were supplemented with erythromycin (12.5 μg/mL), spectinomycin (1 mg/mL), kanamycin (800 μg/mL) and tetracycline (15 μg/mL). LB plates were supplemented with spectinomycin (100 μg/mL) and kanamycin (50 μg/mL).

### General DNA manipulations

All PCR primers used in this study are listed in Supplementary Data 2. PCR reactions were performed with PrimeSTAR HS DNA polymerase (Takara, Shiga, Japan), Taq DNA polymerase (TransGene, Beijing, China), or Phusion^®^ HF DNA polymerase (Thermo Fisher scientific, MA) according to the manufacturers’ instructions. Golden Gate cloning was performed according to the literature procedure^56^. To extract genomic DNAs, 12 mL overnight culture of each strain was centrifuged and washed once with TE (10 mM pH 7.5 Tris-HCl + 1 mM EDTA) buffer. The cells were resuspended in 350 μL TE with 100 μL egg white lysozyme (50 mg/mL, AMRESCO, Shanghai, China) and incubated at 37 °C for three hours, after which 100 μL of 10% SDS was added and the mixture was incubated at 37 °C for ten minutes. The mixture was then acidified with 55 μL of 3 M sodium acetate (pH 5.2) and extracted twice with 300 μL phenol:chloroform:isoamyl alcohol (25:24:1). DNAs were precipitated by addition of an equal volume of isopropanol, washed once with 70% ethanol and dissolved in 100 μL TE buffer after drying.

Natural transformations were performed according to a reported protocol as following^24^. In brief, *S. mutans* strains were cultivated overnight in BHI liquid. On the following day, the stationary phase cultures were diluted 1:20 in THYE and incubated at 37 °C for three hours until the OD_600_ reached 0.2–0.3. The DNA and the competence-stimulating peptide CSP (the final concentration is 1 μg/mL) were added into 500 μL cell culture and incubated for another 3 h. After this, cells were diluted appropriately and plated on selective BHI plates. The plates were incubated at 37 °C until colonies were visible. Forty microgram genomic DNA was used for each transformation.

### DNA synthesis and sequence analyses

Synthesis of DNAs was carried out in Genery Co. (Shanghai, China). Genomic DNA sequencing of *S. mutans* 35 was performed using the Illumina Hiseq 2000 system. The online software antiSMASH (https://antismash.secondarymetabolites.org/#!/start) was used to predict gene clusters and secondary metabolites. A BLASTP search was used to predict protein functions (https://blast.ncbi.nlm.nih.gov/Blast.cgi). A BLASTX search (https://blast.ncbi.nlm.nih.gov/Blast.cgi) was used to analyze the distribution of BGC1 in the NCBI database with the expected threshold of 10. The ribosome binding site sequence analysis was performed with RBS Calculator (https://salislab.net/software/reverse).

### Cloning of BGCs with the CATCH technique

The 5.3-kb pSET154 was constructed from pSET153 by replacing the apramycin resistance gene with kanamycin. The CATCH technique was used to capture the gene clusters according to the described method^28^. Using the cloning of BGC1 as an example, two sgRNA sequences were selected for both regions flanking BGC1. The BGC1-sgRNAF and BGC1-sgRNAR in vitro transcription templates were prepared by overlapping PCR of 3 primers: a primer (BGC1-gF-P or BGC1-gR-P) containing the target sequence, and 2 others (guide RNA-F and guide RNA-R) carrying the crRNA-tracrRNA chimera sequence. Cas9 digestion of *S. mutans* 35 genomic DNA plug using BGC1-sgRNAF and BGC1-sgRNAR was carried out according to the literature procedures^28^. The digested DNA was precipitated with ethanol and suspended in 20 μL DNase-free water. The backbone of the pSET154 vector was amplified from plasmid pSET154 using primers BGC1-p15A-F and BGC1-p15A-R, which include a ~ 30 bp overlap with one end of the target fragment. Fifty nanograms backbone and 1 μg digested genome fragment were assembled using Gibson Assembly Master Mix (NEB, Ipswich, MA). After ligation, the product was transformed into electrocompetent *E. coli* EPI300 cells. Recombinant colonies were screened using primers BG1F/kana-RV and F2d1cxF/BG1R. Plasmid pSET154 containing the BGC1, was named pSET154-1. The mutanobactin gene cluster was cloned with primers mub-gF-P/mub-gR-P and mub-p15A-F/mub-p15A-R; BGC4 was cloned with primers BGC4-gF-P/BGC4-gR-P and BGC4-p15A-F/BGC4-p15A-R; BGC6 was cloned with primers BGC6-gF-P/BGC6-gR-P and BGC6-p15A-F/BGC6-p15A-R as described above. Primers used in this section and the followings were all list in Supplementary Data 2.

### Cloning of BGC1 into *S. mutans* UA159

To eliminate the *Bsa*I site of pFW5 by point mutation, the plasmid was PCR-amplified with primer pair Amp-deBsaF/AmpR-deBsaI and re-ligated by T4 DNA ligase to generate plasmid pNCL, which was then amplified as a 2.7-kb fragment using primer pair pFW5F-mubdnR/pFW5R-mubupF. The 0.8-kb FL fragment at the right end of the mutanobactin gene cluster was amplified with primer pair mubdnF-OLBsaI/mubdnR using a synthesized DNA fragment as template, in which *mubP* was under the control of the constitutive promoter CP25; the 0.9-kb FR fragment at the left end of the mutanobactin gene cluster was amplified from *S. mutans* UA159 genomic DNAs with primer pair mubupF2/mubupR2-OLBsaI. The three amplicons (pNCL fragment, FL, and FR) with overlapping regions were ligated with a one step cloning kit (Vazyme Biotech Co., Ltd, Nanjing, China) according to the manufacturers’ instructions to generate plasmid pNCL-159.

The 2.1-kb IFDC2 cassette for counterselection was amplified using pIFDC2 as a template with primer pair IFDC2-F-BsaI/IFDC2-R-BsaI. The 2.0-kb CAL1 and the 1.8-kb CAR flanking BGC1 were PCR amplified from *S. mutans* 35 genomic DNAs with primer pairs NN2025c2LBupF/NN2025c2LBupR and NN2025c2RBdnF/NN2025c2RBdnR, respectively. The 10.2-kb plasmid pNCL-159/BGC1 was then constructed by insertion of CAL, IFDC2, and CAR into pNCL-159 using the Golden Gate cloning strategy. After introduction of the suicide plasmid pNCL-159/BGC1 into *S. mutans* UA159 via natural transformation, the erythromycin resistant and spectinomycin sensitive transformants were selected as the desired recipient strain *S. mutans* UA159-RS/BGC1, which were verified by PCR with primers mub-F/IFDC2-R and IFDC2-F/mub-R.

Genomic DNAs of *S. mutans* 35 were transformed to the recipient strain *S. mutans* UA159-RS/BGC1 via natural transformation, and the colonies that could grow on BHI plates containing 4 mg/mL *p*-Cl-Phe were selected as positive transformants. The positive transformants that could not grow on BHI plates with erythromycin were PCR-verified using primers orfB-F/mubdnR, bacA-F/bacA-R, orfE-F/orfE-R, and mubupF/orfI-R as the designed *S. mutans* UA159/BGC1 (Supplementary Fig. 1).

### Cloning of BGC1 into *S. mutans* UA159^*^

The two 0.6-kb fragments flanking the *smu2080-2081* operon were amplified from *S. mutans* UA159 genomic DNAs using primer pairs 2080upF/2080UpR-BsaI and 2080dnF-BsaI/2080dnR, respectively. The 0.2-kb *xylA*_*p*_*-xylA*_*o*_ promoter sequence was amplified using pZX10 as a template with primer pair xylApF-BsaI/xylOR-BsaI. The 1.9-kb tetracycline resistance gene *tetM* was amplified using pAM120 as a template with primers tetMF-BsaI/tetMR-BsaI. The four amplicons all have *Bsa*I sites and were ligated by Golden Gate cloning to generate a 3.3-kb fragment, which was transformed to *S. mutans* UA159, and the transformants were selected on BHI plate containing tetracycline (15 μg/mL) to obtain the double crossover recombinant strains *S. mutans* UA159^*^. The genotype of *S. mutans* UA159^*^ was PCR-verified using primer pair 2080upF/2080dnR.

The 1.4-kb kanamycin resistance cassette was amplified from plasmid pTV1-OK with primers kanF-BsaI/kanR-xylR; the 1.3-kb *gyrA*_*p*_*-xylR* fragment was amplified from pZX9 using primer pair gyrApF-BsaI/xylRR-kan. The two fragments have an overlapping region, and they were used as templates for an overlapping PCR with primer pair kanF-BsaI/gyrApF-BsaI to generate a 2.7-kb KRX^o^ cassette. The KRX^o^ cassette was then inserted into pGH by one step cloning kit to generate a 5.6-kb plasmid pKR01. To ensure the efficient expression of XylR, its ribosomal binding site GAGGAGGATAAACAAAGGA was replaced with a stronger one ACGCGACCAGCGCGTCCAAGAAGGAGGAATTAC in *S. mutans* by PCR-amplifying pKR01 with primer pair xylRF-RBSa/gyrpR-RBSa, phosphorylating the ends of the PCR product with T4 polynucleotide kinase and self-ligating the 5.6-kb fragment with T4 DNA ligase to generate plasmid pKR02. Successful construction of pKR02 was verified by DNA sequencing. The 2.7-kb KRX cassette in pKR02 was PCR amplified with primer pair kanF-BsaI/gyrApF-BsaI, and inserted into pNCL-159 together with CAL1 and CAR1 using Golden Gate cloning to generate plasmid pNCL-159/BGC1tet. After introduction of the suicide plasmid pNCL-159/BGC1tet into *S. mutans* UA159^*^ via natural transformation, the kanamycin resistant and spectinomycin sensitive transformants were selected as the desired recipient strain *S. mutans* UA159^*^-RS/BGC1 and verified by PCR with primer pairs mub-F/KR-R, KR-F/mub-R (Supplementary Fig. 18).

Genomic DNAs of *S. mutans* 35 were transformed to the recipient strain *S. mutans* UA159^*^-RS/BGC1 via natural transformation, and the colonies that could grow up on BHI plate containing 15 μg/mL tetracycline were selected as positive transformants. The positive transformants that could not grow up on BHI plates with kanamycin were picked as *S. mutans* UA159^*^/BGC1. The genotype of *S. mutans* UA159^*^/BGC1 was verified by PCR following the same method used for verification of *S. mutans* UA159/BGC1 (Supplementary Fig. 1).

### Cloning of the other BGCs into *S. mutans* UA159^*^

One step cloning of BGC2-BGC6, BGC6a and BGC6b into *S. mutans* UA159^*^ using the NabLC technique were carried out with the same procedure as the cloning of BGC1, except the capture arms used for each BGC were different and the genomic DNAs were from different donor strains. The two capturing arms for BGC2 cloning were amplified from *Sa. epidermidis* ATCC 35984 genomic DNAs using primer pairs Ser-RS11480F-BsaI/Ser-RS11480R-BsaI and Ser-RS11475F-BsaI/Ser-RS11475R-BsaI. The capturing arms for BGC3 were amplified from *Streptococcus equinus* ATCC 33317 with primers 2605-upF-BsaI/2605-upR-BsaI and 2670-dnF-BsaI/2670-dnR-BsaI. The capturing arms for BGC4 were amplified from *S. mutans* NMT4863 genomic DNAs with primer pairs NMT4863C1upF-BsaI/NMT4863C1upR-BsaI and NMT4863C1dnF-BsaI/NMT4863C1dnR-BsaI. The capturing arms for BGC5 were amplified from *Clostridium sporogenes* ATCC 19404 genomic DNAs using primers RS13190-upF-BsaI/RS13180-upR-BsaI and RS13120-dnF-BsaI/RS13115-dnR-BsaI. The two capturing arms for BGC6 cloning were amplified from *S. mutans* MT4653 genomic DNAs using primers MT4653upF-BsaI/MT4653upR-BsaI and MT4653dnF1-BsaI/MT4653dnR1-BsaI. The same left capturing arm was used for BGC6, BGC6a, and BGC6b. For the cloning of BGC6a and BGC6b, the corresponding right capturing arms were amplified using primer pairs MT4653dnF2-BsaI/MT4653dnR2-BsaI (BGC6a) and MT4653dnF3-BsaI/MT4653dnR3-BsaI (BGC6b), respectively. All constructed *S. mutans* UA159^*^-RS/BGC strains were PCR-verified with the same primer pairs mub-F/KR-R, KR-F/mub-R (Supplementary Fig. 18). After the target genomic DNAs were transformed into those recipient strains, positive transformants that could grow on BHI plates containing 15 μg/mL tetracycline were selected. The positive transformants that could not grow on BHI plates with kanamycin were picked and verified as the recombinant strains harboring the desired BGCs (Supplementary Fig. 1).

### Multiple rounds cloning of BGC6 into *S. mutans* UA159^*^

With the 40-kb fragment of BGC6 already cloned into *S. mutans* UA159^*^/BGC6b, we used the NabLC technique to iteratively piece together multiple large DNA fragments and thus reconstruct a large BGC. The remaining 33.7-kb of BGC6 was sequentially cloned as the 23.6-kb BGC6c and the 10.1-kb BGC6d (Supplementary Fig. 2). To clone the 23.6-kb BGC6c, the 0.5-kb FL6c fragment at the right end of BGC6b was amplified with primer pair MT4653upF4/MT4653upR4-BsaI from *S. mutans* UA159^*^/BGC6b genomic DNA. The 2.0-kb CAR6c was amplified with primer pair MT4653dnF4-BsaI/MT4653dnR4-BsaI using *S. mutans* MT4653 genomic DNAs as template. The two fragments were ligated with KRX and FR using Golden Gate cloning to generate the FL6c-KRX-CAR6c-FR fragment, which was transformed to *S. mutans* UA159^*^/BGC6b and screened for kanamycin resistance to obtain the recipient strain *S. mutans* UA159^*^-RS/BGC6c. In *S. mutans* UA159^*^-RS/BGC6c, BGC6b was used as the left capture arm for BGC6c. Genomic DNAs of *S. mutans* MT4653 were then transformed to *S. mutans* UA159^*^-RS/BGC6c and colonies that were tetracycline resistant and kanamycin sensitive were picked as *S. mutans* UA159^*^/BGC6bc harboring a 63.6-kb section of BGC6. The genotype of *S. mutans* UA159^*^/BGC6bc was PCR-confirmed with primer pairs 4653-F4/4653-R4, 4653-F5/4653-R5, 4653-F6/4653-R6. The 10.1-kb BGC6d was cloned with a similar procedure as that for BGC6c. In brief, the 0.6-kb FL6d was amplified with primer pair MT4653upF5/MT4653upR5-BsaI using *S. mutans* UA159^*^/BGC6bc genomic DNAs as template, and the 1.9-kb CAR6d was amplified with primer pair MT4653dnF5-BsaI/MT4653dnR5-BsaI from *S. mutans* MT4653 genomic DNAs. The two fragments were ligated with KRX and FR using Golden Gate cloning to generate the FL6d-KRX-CAR6d-FR fragment, which was transformed to *S. mutans* UA159^*^/BGC6bc and screened for kanamycin resistance to obtain the recipient strain *S. mutans* UA159^*^-RS/BGC6d. Genomic DNAs of *S. mutans* MT4653 were then transformed to *S. mutans* UA159^*^-RS/BGC6d and screened for colonies that were tetracycline resistant and kanamycin sensitive, which were picked as *S. mutans* UA159^*^/BGC6 and verified by PCR with primer pairs 4653-F7/4653-R7, 4653-F8/4653-R8, 4653-F9/4653-R9 (Supplementary Fig. 1).

### Evaluation of different counterselection systems

Background cell numbers of the PheS^*^ based counterselection system was obtained by spreading 10 μL of appropriately diluted *S. mutans* UA159-RS/BGC1 culture on a BHI plate with 4 mg/mL *p*-Cl-Phe. The culture was diluted 10^8^-fold and spread (10 μL) on a blank BHI plate for calculation of the total living cell number. The background rate of the PheS^*^ based counterselection system was then calculated using the background cell number to divide the total living cell number. The background rate of the *tetM* based-counterselection system was similarly obtained, except *S. mutans* UA159^*^-RS/BGC1 was grown on a BHI plate with 15 μg/mL tetracycline for background cell number calculation. The results were obtained from at least three independent experiments.

Screening efficiency comparison of the two selection systems was performed as follows: the same amount (40 μg) of genomic DNAs of *S. mutans* 35 was transformed to the recipient strains *S. mutans* UA159-RS/BGC1 and *S. mutans* UA159^*^-RS/BGC1 respectively, and sixty-four positive transformants were picked in each case. The correct *S. mutans* UA159/BGC1 strains were screened for their sensitivities to erythromycin and verified by PCR as described. The correct *S. mutans* UA159^*^/BGC1 strains were screened for their sensitivities to kanamycin and verified by PCR as described.

### Activation of BGC2 in *S. mutans* UA159^*^

To functionally express BGC2 in *S. mutans* UA159^*^, the constitutive promoter *gyrA*_*p*_ in the IFDC2 cassette was used to control the entire gene cluster. The 2.1-kb IFDC2 cassette (containing promoter *gyrA*_*p*_) was PCR-amplified using pIFDC2 as a template with primer pair ldhF-BsaI/ermR-BsaI. To insert the promoter *gyrA*_*p*_ upstream of BGC2, two 0.8-kb homologous sequences flanking the insertion locus were amplified using primer pairs Ser-11480F2-BsaI/Ser-11480R and mubdnF3-BsaI/mubdnR from *S. mutans* UA159^*^/BGC2 genomic DNAs. The three amplicons were ligated using Golden Gate cloning with IFDC2 in the middle and transformed to *S. mutans* UA159^*^/BGC2. Colonies resistant to erythromycin on BHI plates were selected and PCR-verified using primer pair mubdnR/Ser-11480R as *S. mutans* UA159^*^/*gyrA*_*P*_-BGC2. For production of pyrazinones, *S. mutans* UA159^*^/*gyrA*_*P*_-BGC2 and *S. mutans* UA159^*^-RS/BGC2 (negative control) were incubated in BHI broth statically at 37 °C in sealed bottles for 72 h. After centrifugation, the supernatants were extracted by equal volume of ethyl acetate for three times, evaporated in vacuum and dissolved in methanol for HPLC and LC-MS analyses.

### Activation of BGC1 in *S. mutans* UA159^*^

BGC1 was activated by inserting the xylose-inducible promoter *xylS1*_*P*_ upstream of this gene cluster via homologous recombination. The 1.4-kb DNA fragment containing the *xylS1*_*P*_ promoter was amplified from pZX9 using primer pair xylOR-BsaI/xylRR-kan. The 1.4-kb fragment containing the kanamycin resistance gene was amplified using plasmid pTV1-OK as a template with primer pair kanF-BsaI/kanR-xylR. The two fragments have an overlapping region, so they were mixed together and used as template for an overlapping PCR with primer pair kanF-BsaI/xylOR-BsaI to generate a 2.8-kb fragment. Meanwhile, a 0.8-kb fragment and a 0.6-kb fragment flanking the insertion locus were PCR cloned with primers mubdnF4-BsaI/mubdnR and orfA-dnF3-BsaI/orfA-dnR from *S. mutans* UA159^*^/BGC1 genomic DNAs. The three amplicons were ligated using Golden Gate cloning with the fragment containing both the kanamycin resistance gene and *xylS1*_*P*_ at the middle and transformed to *S. mutans* UA159^*^/BGC1. Colonies resistant to kanamycin on BHI plates were selected and PCR-verified using primer pair mubdnR/orfA-dnR as *S. mutans* UA159^*^/*xylS1*_*P*_-BGC1. To activate BGC1, *S. mutans* UA159^*^/*xylS1*_*P*_-BGC1 was cultured in ASS broth (pH 5.0) with 0.4% xylose statically at 37 °C in sealed bottles for 72 h. *S. mutans* UA159^*^-RS/BGC1 cultured under the same conditions and *S. mutans* UA159^*^/*xylS1*_*P*_-BGC1 in ASS broth without xylose were used as negative controls. After centrifugation, the supernatants were extracted three times with an equal volume of ethyl acetate, evaporated under vacuum and dissolved in methanol for HPLC and LC-MS analyses.

### Activation of BGC4 in *S. mutans* UA159^*^

BGC4 in *S. mutans* UA159^*^ was activated by inserting two constitutive promoters *gyrA*_*p*_ and *ldh*_*p*_ to replace the transcriptional regulatory gene *orfD* via two rounds of homologous recombination (Supplementary Fig. 19a). The 1.4-kb DNA fragment containing the *gyrA*_*p*_ and *ldh*_*p*_ promoters was amplified from pZX9 using primer pair xylOR-BsaI/xylRR-kan. The 1.4-kb fragment containing the kanamycin resistance gene was amplified from plasmid pTV1-OK with primer pair kanF-BsaI/kanR-xylR. The two fragments have an overlapping region facilitating their assembly via overlap PCR with primer pair kanF-BsaI/xylOR-BsaI to generate a 2.8-kb fragment. The 0.6-kb fragment and 0.5-kb fragment flanking the insertion locus were PCR cloned with primers orfC-F/orfC-R-BsaI and orfE-F-BsaI/orfE-R from *S. mutans* UA159^*^/BGC4 genomic DNAs. The three amplicons were ligated using Golden Gate cloning and transformed to *S. mutans* UA159^*^/BGC4. Colonies resistant to kanamycin on BHI plates were selected and PCR-verified as *S. mutans* UA159^*^/*xylS1*_*P*_-BGC4. Next, the kanamycin resistance gene and *xylR* gene were removed to create constitutive promoters. The 0.6-kb fragment and 0.7-kb fragment flanking the kanamycin resistance gene and *xylR* gene were PCR amplified with primers orfC-F/orfC-R2-BsaI and gyrA-F-BsaI/orfE-R. The two fragments were ligated using Golden Gate cloning and transformed to *S. mutans* UA159^*^/*xylS1*_*P*_-BGC4. Colonies resistant to tetracycline on BHI plates were selected and PCR-verified as *S. mutans* UA159^*^/*ldh*_*P*_-BGC4. For the production of SNC1-465, *S. mutans* UA159^*^/*ldh*_*P*_-BGC4 and *S. mutans* UA159^*^-RS/BGC4 (negative control) were incubated in ASS broth statically at 37 °C in sealed bottles for 24 h. After centrifugation, the supernatants were extracted three times with equal volumes of ethyl acetate, evaporated in vacuum, and dissolved in methanol for HPLC and LC-MS analyses.

### Construction of *S. mutans* UA159^*^/*ldh*_*P*_-BGC4 ∆*orfF*

The *orfF* gene in-frame deletion mutant was constructed via two rounds of homologous recombination (Supplementary Fig. 19b). The 0.7-kb fragment and 0.6-kb fragment flanking the *orfF* gene were PCR amplified with primers NMT4863C1de-upF1/NMT4863C1de-upR1-BsaI and NMT4863C1de-dnF1-BsaI/NMT4863C1de-dnR1. The two fragments and KRX cassette were ligated using Golden Gate cloning and transformed to *S. mutans* UA159^*^/*ldh*_*P*_-BGC4. Colonies resistant to kanamycin on BHI plates were selected and PCR-verified. The KRX cassette was then removed as described above. The 0.7-kb fragment and 0.6-kb fragment were PCR amplified with primers NMT4863C1de-upF1/NMT4863C1de-upR2-BsaI and NMT4863C1de-dnF2-BsaI/NMT4863C1de-dnR1, ligated, and transformed to replace the KRX cassette. Colonies resistant to tetracycline on BHI plates were selected and PCR-verified as *S. mutans* UA159^*^/*ldh*_*P*_-BGC4 ∆*orfF*.

### Isolation and synthesis of mutanocyclin

Strain *S. mutans* UA159^*^/*xylS1*_*P*_-BGC1 was inoculated into ASS medium with 0.4% xylose and cultured statically at 37 °C for 72 h. After centrifugation, 12 L supernatant was extracted with two equal volumes of ethyl acetate, concentrated under vacuum and resuspended in 10 mL methanol, which was loaded onto a C18 flash column (40–63 μm, 25 mm × 165 mm) and eluted with 240 mL of 25% methanol and collected in 30 mL fractions. The fractions containing mutanocyclin were concentrated and further purified by semi-preparative HPLC (Zorbax SB-C18, 5 μm, 9.4 mm × 250 mm, Agilent, Santa Clara, CA, USA) eluted with a gradient of acetonitrile in water with 0.1% formic acid (5% for 5 min, 5–100% for 30 min, 100% for 10 min) at a flow rate of 3.5 mL/min. Finally, the sample was refined by semi-preparative HPLC with the same Zorbax SB-C18 column and eluted with a solvent gradient from 20 to 60% acetonitrile with 0.1% formic acid in 30 min at a flow rate of 3.5 mL/min. Synthesis of mutanocyclin from *Boc* protected *D*-Leu was performed according to the literature procedures^57,58^.

### Production of mutanocyclin in *S. mutans* isolates

To obtain *S. mutans* strains with BGC1, all clinically isolated *S. mutans* strains collected in our lab were checked by PCR with degenerate primers KS-F/KS-R for the KS domain of PKS and NRPS-F/NRPS-R for the A domain of NRPS. The amplicons were ligated into the pMD19-T vector, sequenced and aligned with BGC1. Candidate strains were further verified by BGC1 specific primer pairs MutB-F/MutB-R, MutD-F/MutD-R, MutE-F/MutE-R, and MutI-F/MutI-R and confirmed by sequencing. The four *S. mutans* strains were cultured in ASS broth (pH 5.0) statically at 37 °C in sealed bottles for 72 h. Detection of mutanocyclin in the four wild-type strains was performed with HPLC and LC-MS with the chemically synthesized standard as a positive control. Reutericyclin production was checked with HR-LC-MS.

### Isolation of SNC1-465

*S. mutans* strain UA159^*^/*ldh*_*P*_-BGC4 was inoculated into ASS medium (pH 7.0) and cultured statically at 37 °C for 24 h. After centrifugation, 300 L supernatant was extracted with equal volumes of ethyl acetate twice, concentrated under vacuum, and resuspended in about 30 mL methanol. The solution was separated using a Sephadex LH20 column (2.0 cm × 80 cm) and eluted with methanol (with 5% DMSO). Each 15 mL eluted fraction was detected by HPLC and the fractions containing SNC1-465 were concentrated. The concentrated fractions were further purified by semi-preparative HPLC (Zorbax SB-C18, 5 μm, 9.4 mm × 250 mm, Agilent, Santa Clara, CA, USA) eluted with a gradient of acetonitrile (with 5% DMSO) in water (with 0.1% formic acid and 5% DMSO) (65% for 16 min, 65%-100% for 5 min, 100% for 6 min) at a flow rate of 3.5 mL/min. Finally, the sample was refined by semi-preparative HPLC with the same Zorbax SB-C18 column and eluted with a solvent gradient from 82 to 100% methanol (with 5% DMSO) in water (with 0.1% formic acid and 5% DMSO) in 20 min at a flow rate of 2.5 mL/min.

### Marfey’s analysis of SNC1-465

Marfey’s analysis was performed following the procedure reported by Guo et al.^59^. In brief, About 1.0 mg of SNC1-465 was hydrolyzed with 200 μL of 6 N HCl at 110 °C for 5 h. The acid hydrolysate was dried and dissolved in 100 μL of 0.1 N HCl. To 50 μL of the acidic solution, 80 μL of 1 N NaHCO_3_ and 400 μg 1-fluoro-2,4-dinitrophenyl-5-*L*-alanine amide (FDAA, Marfey’s reagent, J&K Chemical, Beijing, China) with 40 μL acetone was added, and the mixture was heated at 50 °C for 0.5 h. The reaction mixture was cooled to room temperature, neutralized with 40 μL 1 N HCl, and dried. The residue was dissolved in 50 μL of acetonitrile and the FDAA derivative solution was analyzed by HPLC. The FDAA derivatives of the SNC1-465 acid hydrolysate were identified by comparing the retention times with FDAA derivatized standard amino acids.

### Antibacterial assay

Antibacterial activity was measured by the micro-broth dilution method in 96-well culture plates according to the Standard of National Committee for Clinical Laboratory^60^. Erythromycin and tetracycline (Jiangchen Yuanyuan Biotechnology Co., Beijing, China) were used as a positive control. The tested bacteria *Escherichia coli* JM109, *Bacillus subtilis* BS 168, *Streptococcus sanguinis* NY101, *Streptococcus oralis* 10557, *Streptococcus gordonii* 10556, *Lactococcus Lactis* MG1363, *Veillonella atypica* PK1910 were incubated in BHI broth (*Veillonella atypica* PK1910 in BHI supplemented with 0.6% sodium lactate); the *Paenibacillus larvae* ATCC 13537 was incubated in MPYGP-0.01% thiamine medium, at 37 °C for 12 h, and the cell concentration was diluted to approximately 1 × 10^6^ CFU with BHI broth or MPYGP-0.01% thiamine medium. The bacteria were then incubation at 37 °C for 24 h and the MICs were read.

### Matrigel plug assay

Animal experiments were conducted in accordance with the protocols approved by the Institutional Animal Care and Use Committee (IACUC) of University of Massachusetts Amherst. Briefly, 0.25 mL growth factor-reduced Matrigel (BD Biosciences, San Jose, CA), which was pre-mixed with mutanocyclin or DMSO vehicle, was subcutaneously injected into 6-week-old C57BL/6 male mice in the abdominal area. After 5 days, the mice were euthanized to dissect the implanted Matrigel plugs. The plugs were digested using Corning® cell recovery solution (Corning, NY), filtered through 70 μm cell sorters (BD Biosciences, San Jose, CA) to obtain single cell suspension, which were stained with FITC-conjugated anti-mouse CD45 antibody (catalog number: 103107; clone: 30-F11, dilution ratio: 1:500) and isotype control antibody (catalog number: 400605; clone: RTK4530, dilution ratio: 1:500, BioLegend, San Diego, CA). The stained cells were analyzed using BD LSRFortessa™ cell analyzer (BD Biosciences, San Jose, CA), and data were analyzed using FlowJo software (FlowJo LLC, Ashland, OR). Gating and cell identification strategies are as follows: cell doublets and clumps were eliminated using FSC-H vs. FSC-A gating, and debris was eliminated using FSC-A vs. SSC-A. Dead cells were gated out using Zombie Violet™ dye. In our analyses, leukocytes were identified as CD45^+^ cells.

### Tyrosinase inhibition assay of SNC1-465

The mushroom tyrosinase (Solarbio Technology Co., Beijing, China) inhibition activity of SNC1-465 was measured using *L*-3,4-dihydroxyphenylalanine (*L*-DOPA, Solarbio Technology Co., Beijing, China) as substrate according to published protocol^61^. SNC1-465 was dissolved in DMSO (10 mM) and then diluted to different concentrations. Mushroom tyrosinase and *L*-DOPA were prepared by dissolving in 50 mM Na_2_HPO_4_-NaH_2_PO_4_ buffer (pH6.8). The mixture containing 165 μL of phosphate buffer, 20 μL of 3.6 mM *L*-DOPA, and 5 μL of SNC1-465 at different concentrations (3.9–500 μM) in 96 well microtiter plates were pre-incubated at 30 °C for 10 min. Afterward, 10 μL of tyrosinase solution (about 300 units/mL) was added and incubated for another 15 min. DMSO lacking the test compounds was used as a control, and kojic acid (Solarbio Technology Co., Beijing, China) was used as a positive control. The absorbance was monitored by observing dopachrome formation at 475 nm by a microplate reader (Tecan, Männedorf, Switzerland). The inhibitory effects of the tested compounds were expressed by IC_50_, the concentration that inhibited 50% of the enzyme activity. The inhibitory activity was calculated according to Eq. 1.

1 $$\% \;{\mathrm{inhibition}} = \left[ {1 - \left( {{S} - {B}} \right){/C} - {B}} \right] \times 100$$

*S* is the absorbance of SNC1-465 or kojic acid, *B* is the absorbance of blank, *C* is the absorbance of the control. Each assay was performed three times.

### Spectroscopic analysis

HPLC detection of pyrazinones produced by BGC2 was carried out on a Shimadzu HPLC system (Shimadzu, Kyoto, Japan) using a C18 column (4.6 × 250 mm, 5 μm, Apollo, Alltech, Lexington, Kentucky, USA) under gradient elution conditions as described^38^. The detection wavelength was 325 nm. HPLC detection of mutanocyclin was carried out using the same C18 column on a Shimadzu HPLC system but with a different gradient elution program. The column was developed with solvent A (H_2_O with 0.1% (v/v) formic acid) and acetonitrile at a flow rate of 1.0 mL/min. The percentage of acetonitrile was kept at 5% over 0–5 min, changed from 5 to 40% over 5–25 min and from 40 to 100% over 25–40 min. The detection wavelength was 280 nm. HPLC detection of SNC1-465 was performed similarly as mutanocyclin but the detection wavelength was 210 nm. Marfey’s HPLC analysis was performed using the same C18 column on a Shimadzu HPLC system and the column was developed with solvent A (H_2_O with 0.1% (v/v) formic acid) and solvent B (acetonitrile) at a flow rate of 1.0 mL/min. The percentage of acetonitrile was kept at 20% over 0–5 min, changed from 20 to 60% over 5–35 min and from 60 to 100% over 35–40 min. The detection wavelength was 340 nm. Optical rotation measurement was performed with Anton Paar MCP 200 instrument (Anton Paar GmbH, Anton Paar Strasse, Graz, Austria) at 27 °C. LC-MS analyses were performed on an Agilent 1260/6460 Triple-Quadrupole LC/MS system (Santa Clara, CA) with an electrospray ionization source. HR-ESI-MS was performed on an Agilent 1260 HPLC/6520 QTOF-MS instrument (Santa Clara, CA). NMR spectra were recorded on a Bruker-500 NMR spectrometer (Billerica, MA).

### Chemical characterization of mutanocyclin

The naturally produced mutanocyclin, HR-ESI-MS(+) *m/z* 198.1124 [M + H]^+^ (calculated for C_10_H_15_NO_3_, 198.1125, [M + H]^+^), see Supplementary Fig. 5b;$[\alpha ]_D^{27}$= + 71.98^o^ (C = 0.1, ethanol); ^1^H NMR data, see Supplementary Tables 3 and 4; ^1^H NMR spectra, see Supplementary Fig. 5c.

The chemically synthesized mutanocyclin, Red powder; HR-ESI-MS(+) *m/z* 198.1125 [M + H]^+^ (calculated for C_10_H_15_NO_3_, 198.1125, [M + H]^+^), see Supplementary Fig. 7b; $[\alpha ]_D^{27}$= + 98.88^o^ (C = 0.1, ethanol); ^1^H and ^13^C NMR data, see Supplementary Table 5; ^1^H and ^13^C NMR spectra, see Supplementary Figs. 7c and 8a; ^1^H-^1^H COSY, HMQC and HMBC spectra, see Supplementary Figs. 8b, 9a and 9b.

### Chemical characterization of SNC1-465

HR-ESI-MS(+) *m/z* 465.2761 [M + H]^+^ (calculated for C_28_H_36_N_2_O_4_, 465.2753, [M + H]^+^), see Supplementary Fig. 12b; ^1^H and ^13^C NMR data, see Supplementary Table 6; ^1^H and ^13^C NMR spectra, see Supplementary Figs. 12c and 13a; ^1^H-^1^H COSY, HMQC and HMBC spectra, see Supplementary Figs. 13b, 14a and 14b.

### Reporting summary

Further information on research design is available in the Nature Research Reporting Summary linked to this article.

## Supplementary information

Supplementary Information

Reporting Summary

Description of Additional Supplementary Files

Supplementary Data 1

Supplementary Data 2

Source Data file

## Data Availability

Data supporting the findings of this work are available within the paper and its Supplementary Information files. A reporting summary for this Article is available as a Supplementary Information file. The datasets generated and analyzed during the current study are available from the corresponding author upon request. The BGCs cloned in this study have been deposited into GenBank under the accession numbers MK144293 (BGC1), NC_002976.3 (BGC2), NZ_AUZG00000000.1 (BGC3), AHRZ00000000.1 (BGC4), NZ_LFPM00000000.1 (BGC5), and MK144294 (BGC6). *S. mutans* 35 genome sequence has been deposited into GenBank with the accession number SZVN00000000. The source data underlying Supplementary Figs. 1 and 18 are provided as a Source Data file.
