# Supplementary material for: An anaerobic bacterium host system for heterologous expression of natural product biosynthetic gene clusters
Source: Nat Commun. 2019 Aug 14;10:3665. doi: 10.1038/s41467-019-11673-0 (PMC6694145; doi:10.1038/s41467-019-11673-0)
Supplement: Supplementary file 1 — Supplementary Information [file 41467_2019_11673_MOESM1_ESM.pdf]

An anaerobic bacterium host system for heterologous expression of natural product biosynthetic gene clusters

Hao *et al.*

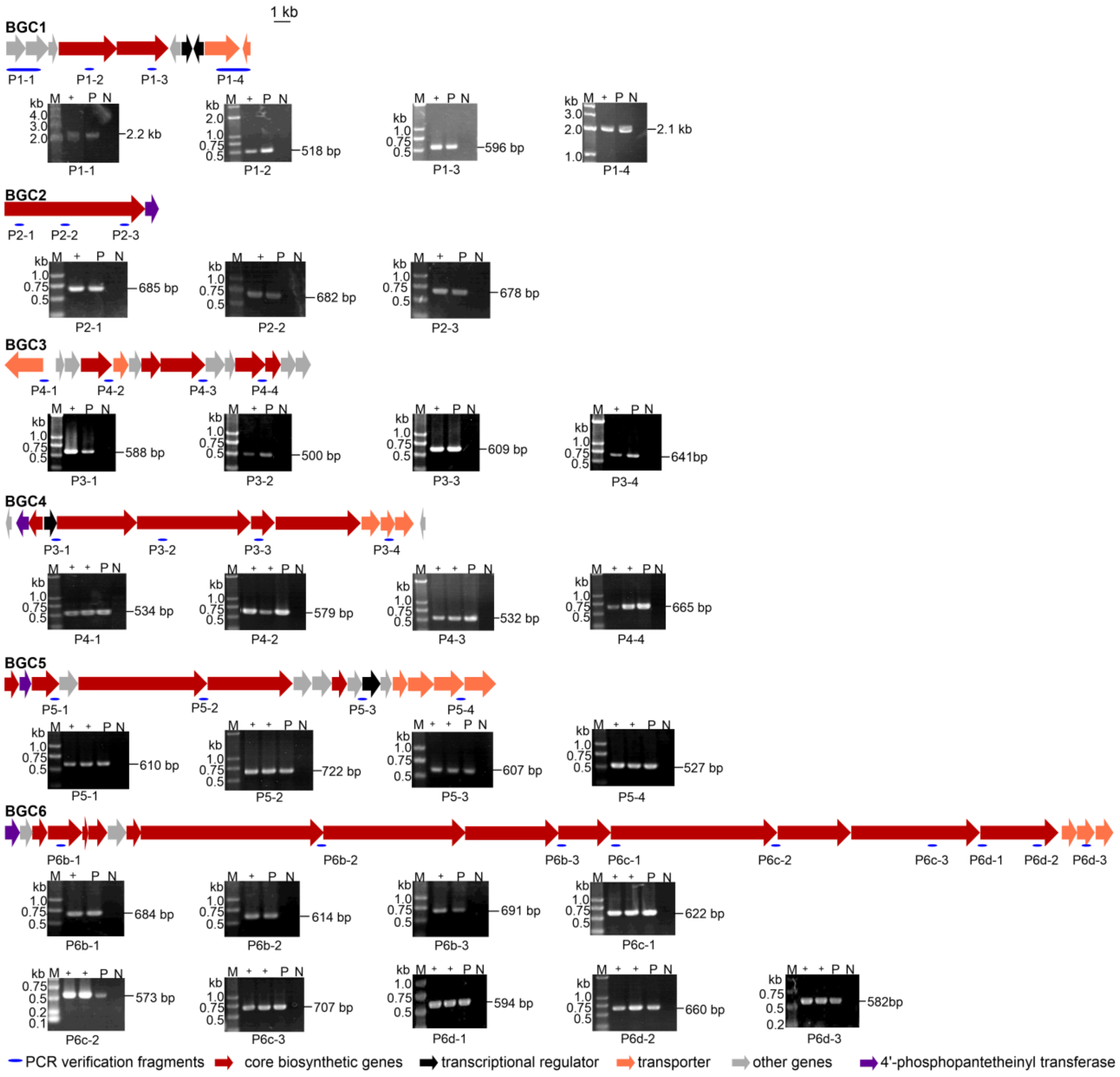

Supplementary Figure 1. PCR verifications of the cloned BGCs in this study. Most BGCs were cloned several times or into more than one recombinant strain. Here we only showed one set of representative PCR verification data. Lane M, DNA marker; Lane P, positive controls using the genomic DNAs of the donor strains as PCR templates; Lane N, negative controls using the genomic DNAs of the recipient strains as PCR templates; Lane +, correct *S. mutans* UA159* recombinant strains with desired BGCs. Source data are provided as a Source Data file.

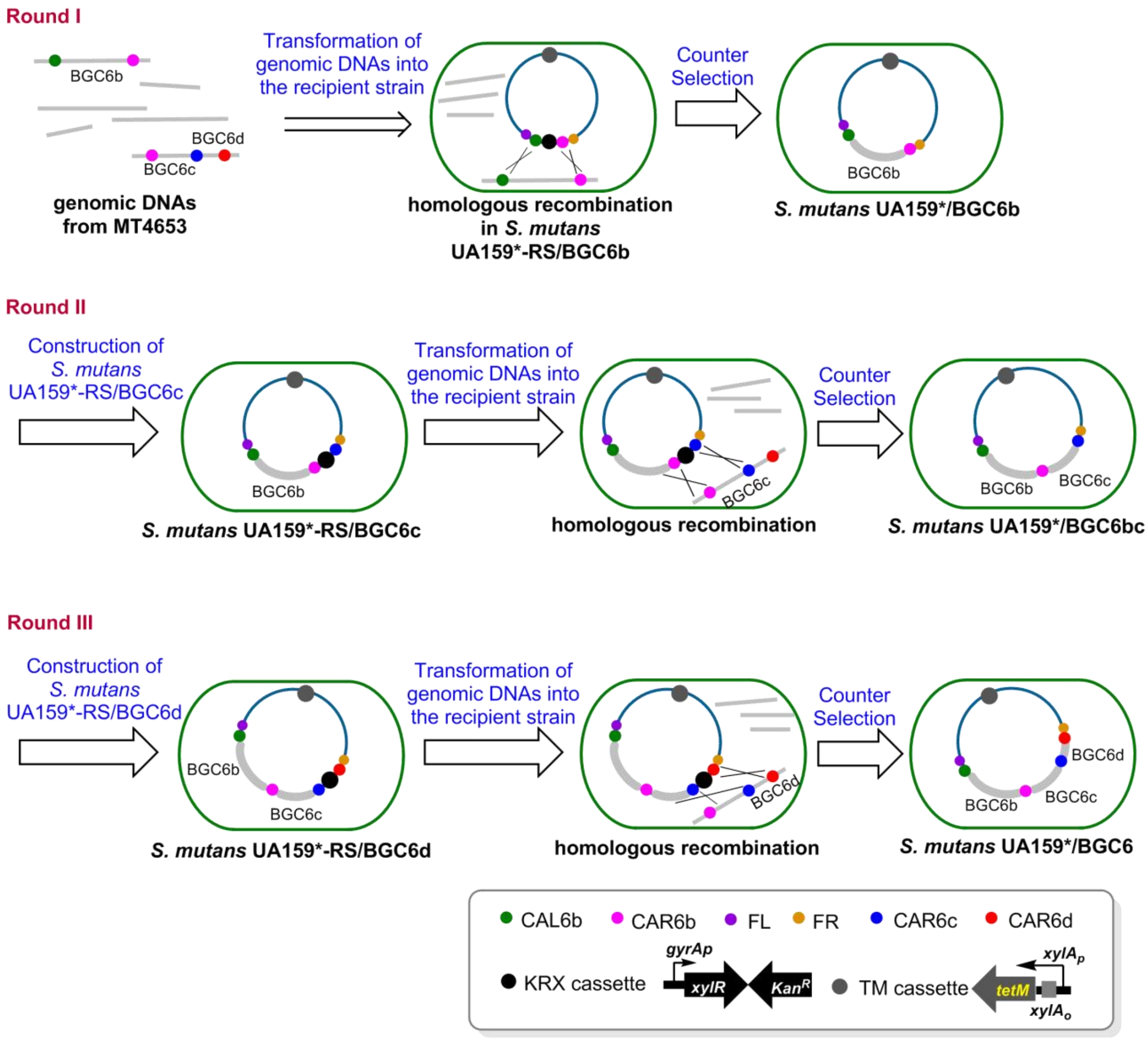

Supplementary Figure 2. Multiple rounds cloning of BGC6 using the NabLC technique in *S. mutans* UA159*. The BGC6 was pieced together with three fragments (BGC6b, BGC6c, and BGC6d) on *S. mutans* UA159* genome in three rounds of cloning with the NabLC technique. Round I, the 40.0-kb BGC6b was cloned in a similar way to the cloning of BGC1; Round II, a recipient strain for BGC6c was constructed based on *S. mutans* UA159*/BGC6b. In *S. mutans* UA159*-RS/BGC6c, the KRX cassette and the right capture arm were put at the right end of BGC6b on the genome. BGC6b was used as the left capture arm. BGC6c was then integrated into the genome of the recipient strain via homologous recombination to generate *S. mutans* UA159*/BGC6bc; Round III, a recipient strain for BGC6d was constructed based on *S. mutans* UA159*/BGC6bc. In *S. mutans* UA159*-RS/BGC6d, the KRX cassette and the right capture arm were put at the right end of BGC6c on the genome. BGC6c was used as the left capture arm. BGC6d was then integrated into the genome of the recipient strain via homologous recombination to generate *S. mutans* UA159*/BGC6 with the whole BGC6.

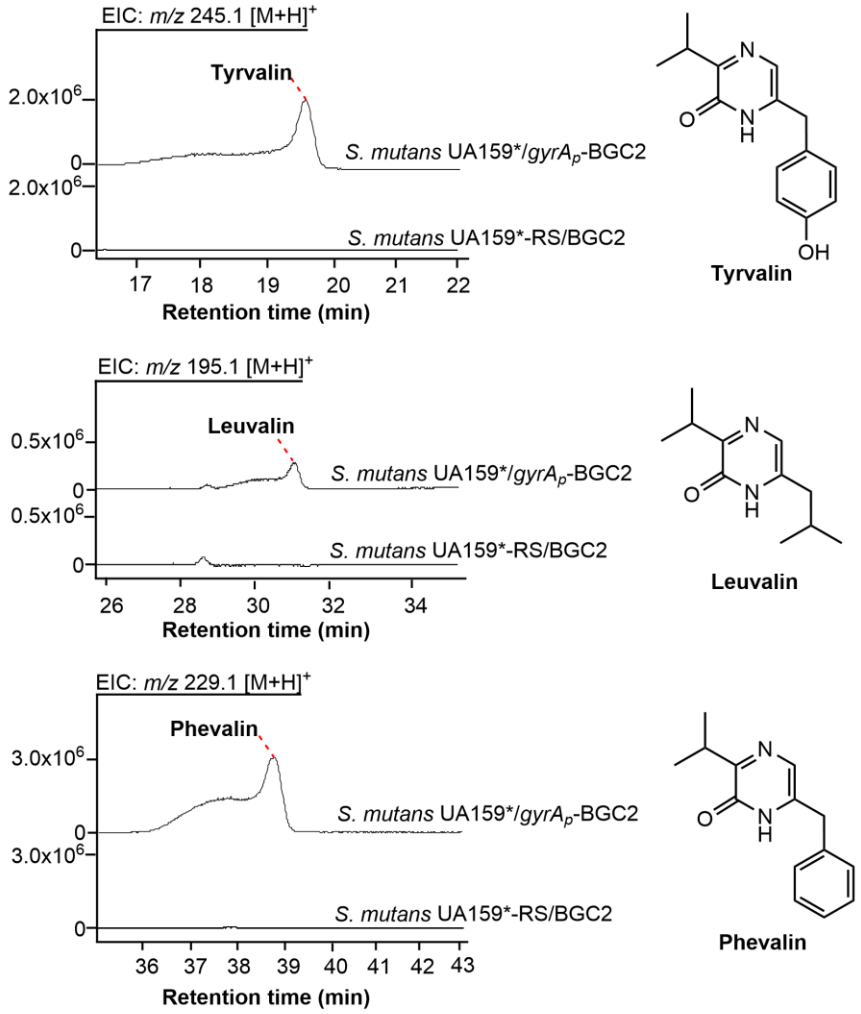

Supplementary Figure 3. LC-MS chromatograms of the three pyrazinones produced by *S. mutans* UA159*/*gyrA*$_P$-BGC2. The supernatant extracts of *S. mutans* UA159*-RS/BGC2 were used as negative controls. EIC, extracted ion count.

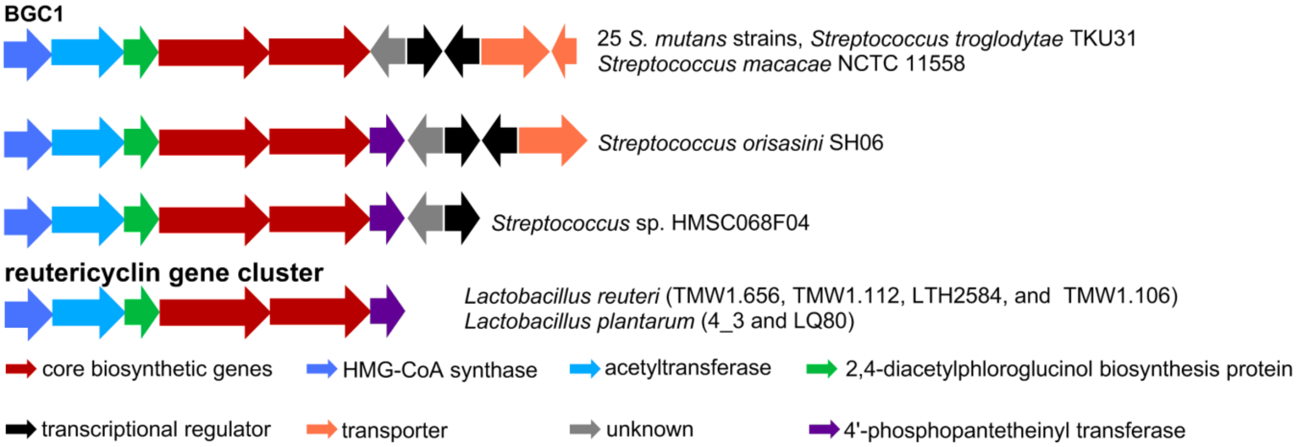

Supplementary Figure 4. The biosynthetic gene clusters analogous to BGC1. Database searching showed that the gene clusters having the same organization as BGC1 were found in 25 *S. mutans* strains and two other *Streptococcus* strains, *S. troglodytae* TKU31 and *S. macacae* NCTC 11558. The reutericyclin biosynthetic gene cluster was observed in six *Lactobacillus* strains. Two more gene clusters similar to BGC1 were found in *Streptococcus orisasini* SH06 and *Streptococcus* sp. HMSC068F04.

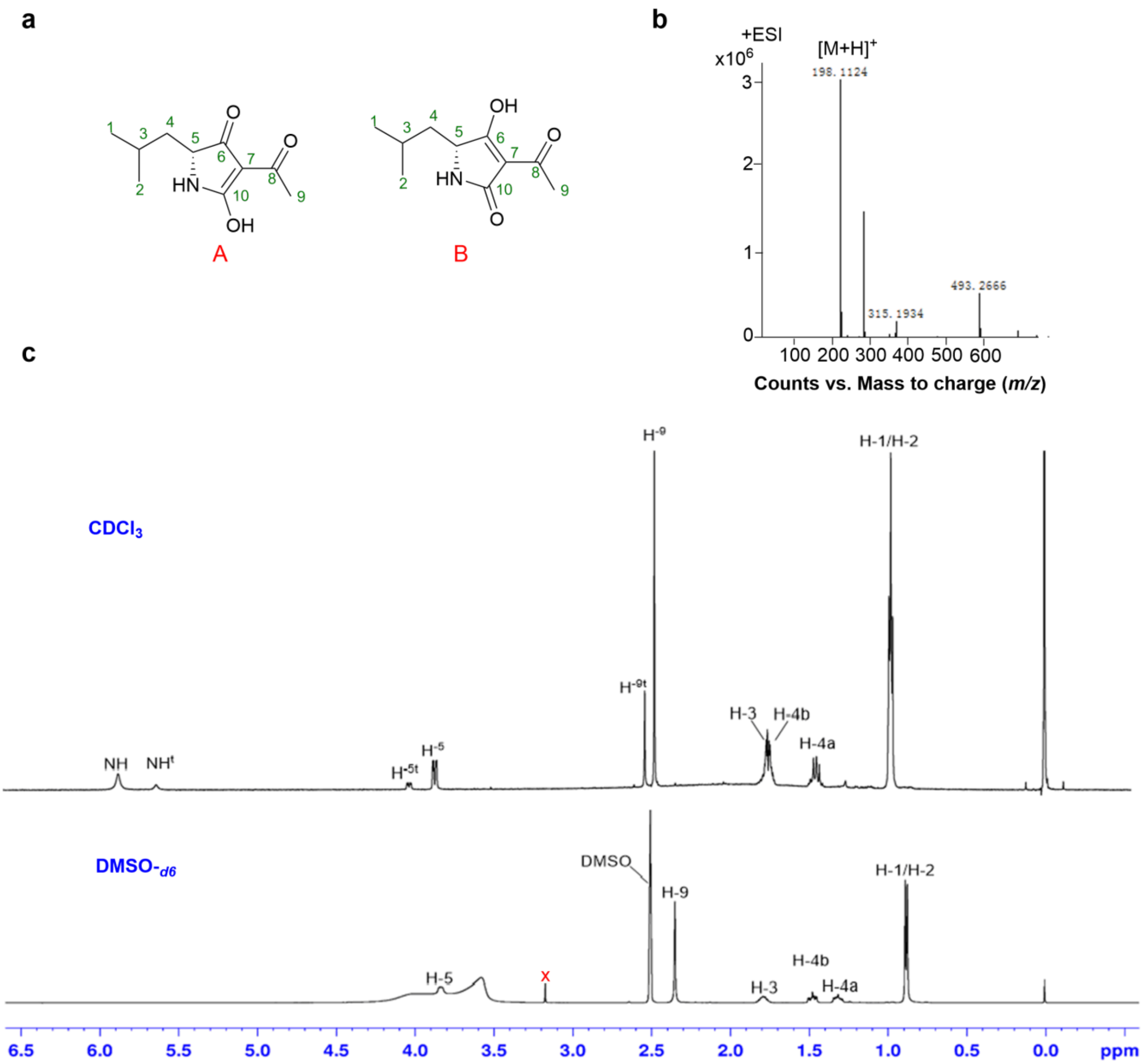

Supplementary Figure 5. Spectral data of mutanocyclin isolated from *S. mutans* UA159*/*xylS1$_P$*-BGC1. (a) Structure of mutanocyclin. A and B are two tautomers of mutanocyclin. **(b)** HR-ESI-MS spectrum of mutanocyclin. **(c)** ^{1}H NMR spectra of mutanocyclin in $CDCl_3$ and in DMSO-$_{d6}$. In DMSO-$_{d6}$, only the A-type tautomer of mutanocyclin was detected. Therefore, NMR comparison of mutanocyclin with its synthesized standard was carried out in DMSO-$_{d6}$.

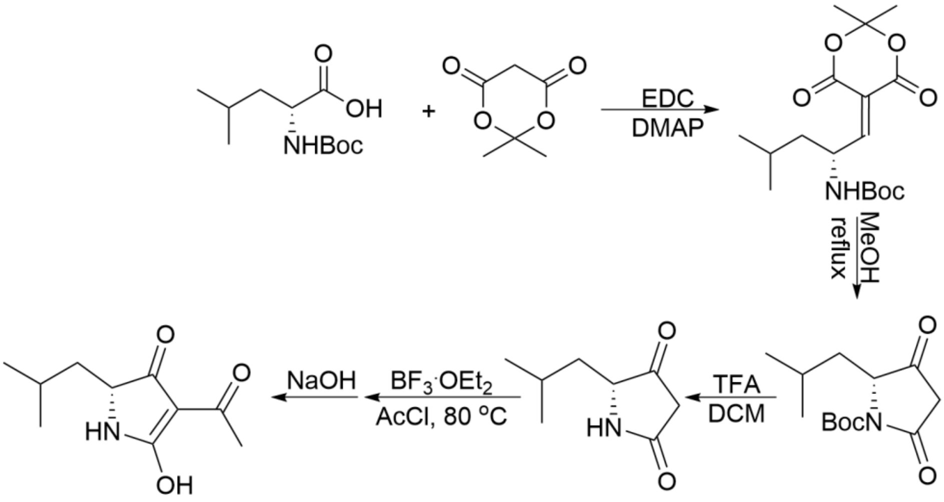

Supplementary Figure 6. Chemical synthesis of mutanocyclin from Boc protected *D*-Leu[1,2]. Boc-*D*-Leu was coupled with Meldrum's acid in the presence of 1-(3-dimethylaminopropyl)-3-ethylcarbodiimide (EDC) hydrochloride and dimethylaminopyridine (DMAP). Cyclization of the lactam ring was achieved by heating in MeOH to provide *N*-protected tetramic acid. After removing the Boc group by TFA, C3-acetylation to generate mutanocyclin was performed with acetyl chloride (AcCl) in the presence of boron trifluoride-diethyl ether complex ($BF_3{\cdot}OEt_2$).

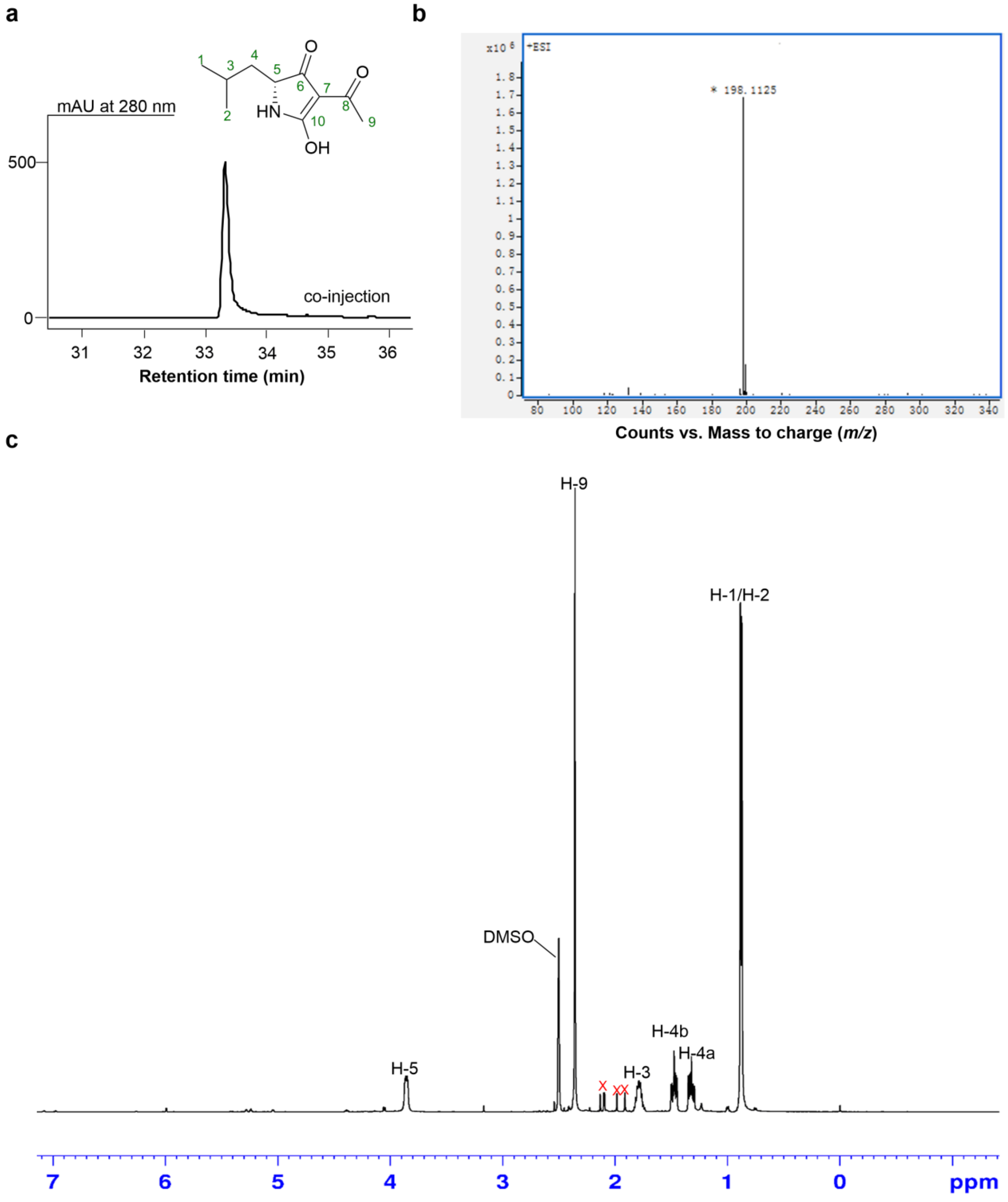

Supplementary Figure 7. Spectral data of chemically synthesized mutanocyclin. (a) HPLC co-injection of the naturally produced mutanocyclin and the chemically synthesized mutanocyclin. **(b)** HR-ESI-MS spectrum of chemically synthesized mutanocyclin. **(c)** ^{1}H NMR spectrum of chemically synthesized mutanocyclin in DMSO-$_{d6}$.

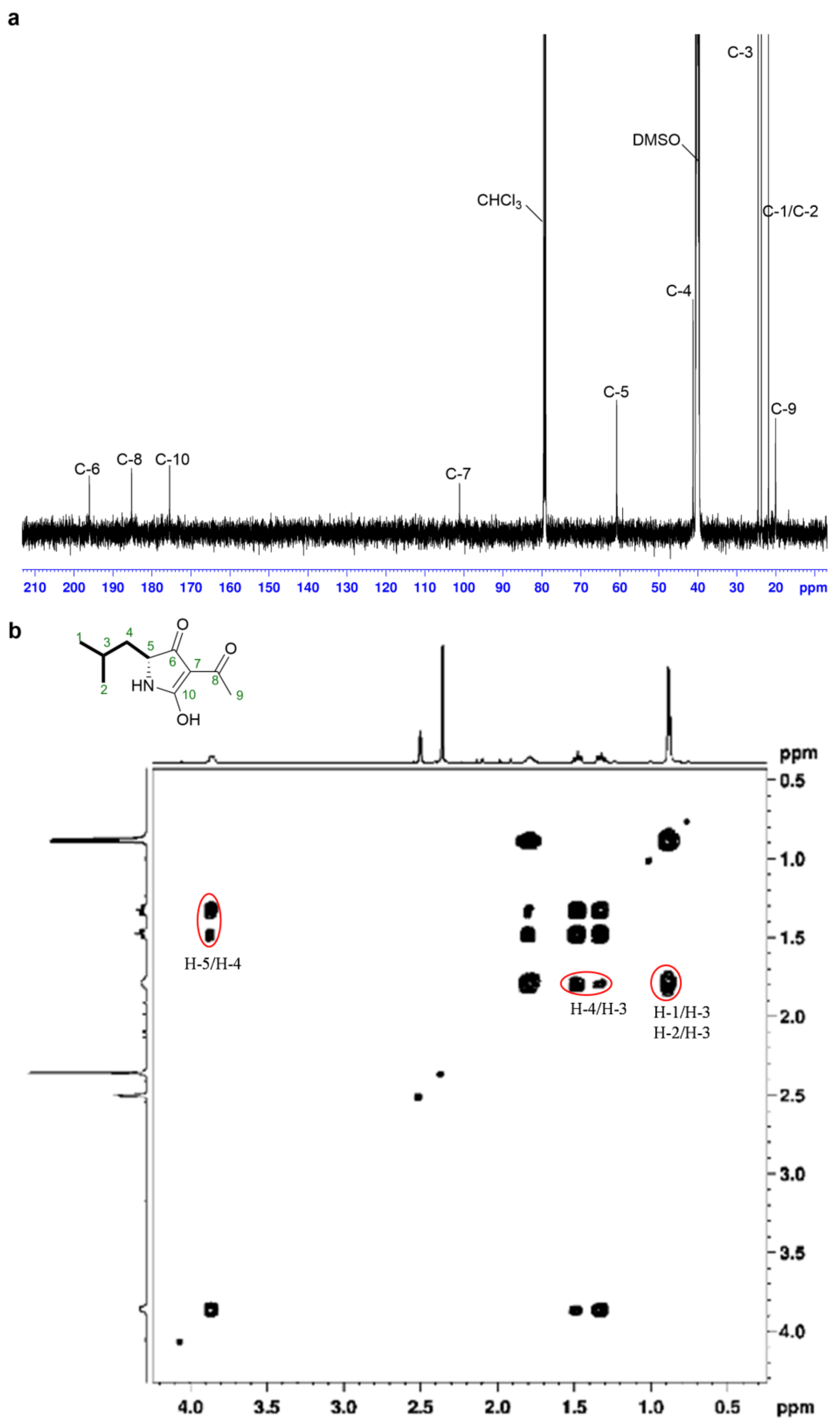

Supplementary Figure 8. Spectral data of chemically synthesized mutanocyclin. (a) ^{13}C NMR spectrum of chemically synthesized mutanocyclin in DMSO-$_{d6}$. A small drop of $CDCl_3$ was added for higher quality acquisition of the C-6, C-8, and C-10 signals. **(b)** ^{1}H-^{1}H COSY spectrum of chemically synthesized mutanocyclin in DMSO-$_{d6}$. The key ^{1}H-^{1}H COSY correlations are marked on the structure with bold lines and in the spectrum with red circles.

a

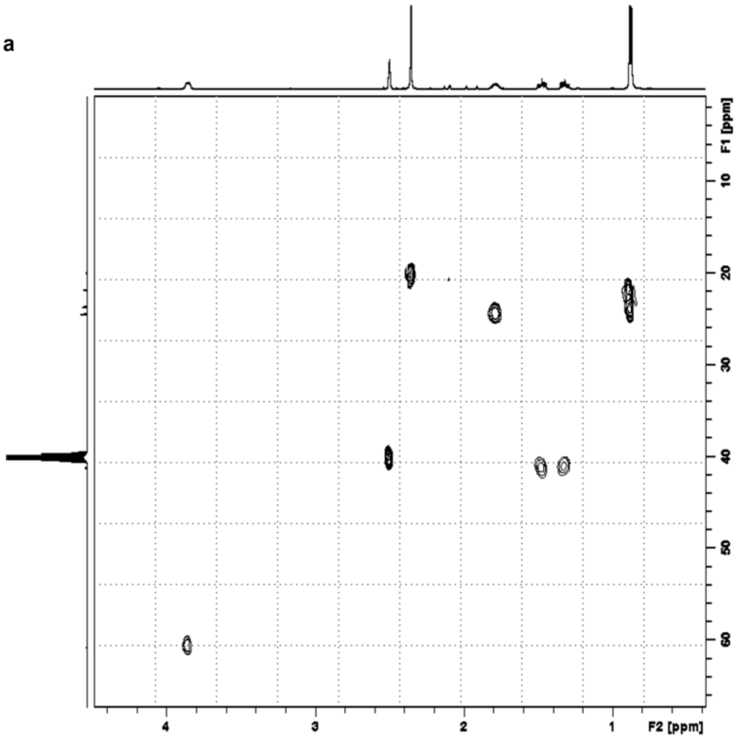

b

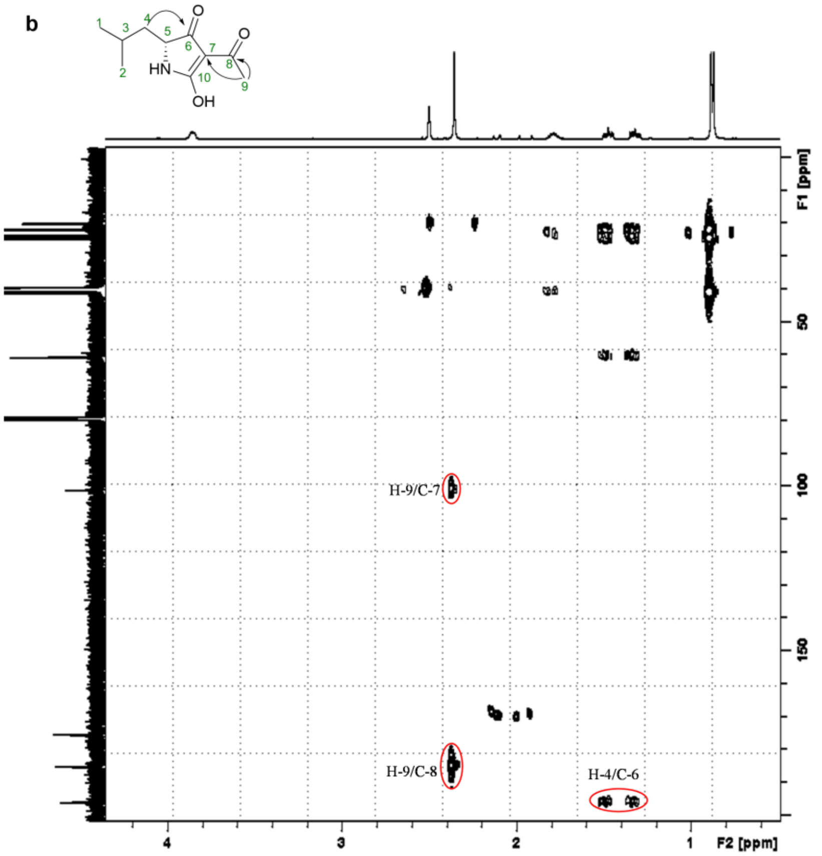

Supplementary Figure 9. Spectral data of chemically synthesized mutanocyclin. (a) HSQC spectrum of chemically synthesized mutanocyclin in DMSO-$_{d6}$. **(b)** HMBC spectrum of chemically synthesized mutanocyclin in DMSO-$_{d6}$. The key HMBC correlations are marked on the structure with arrows and at the spectrum with red circles.

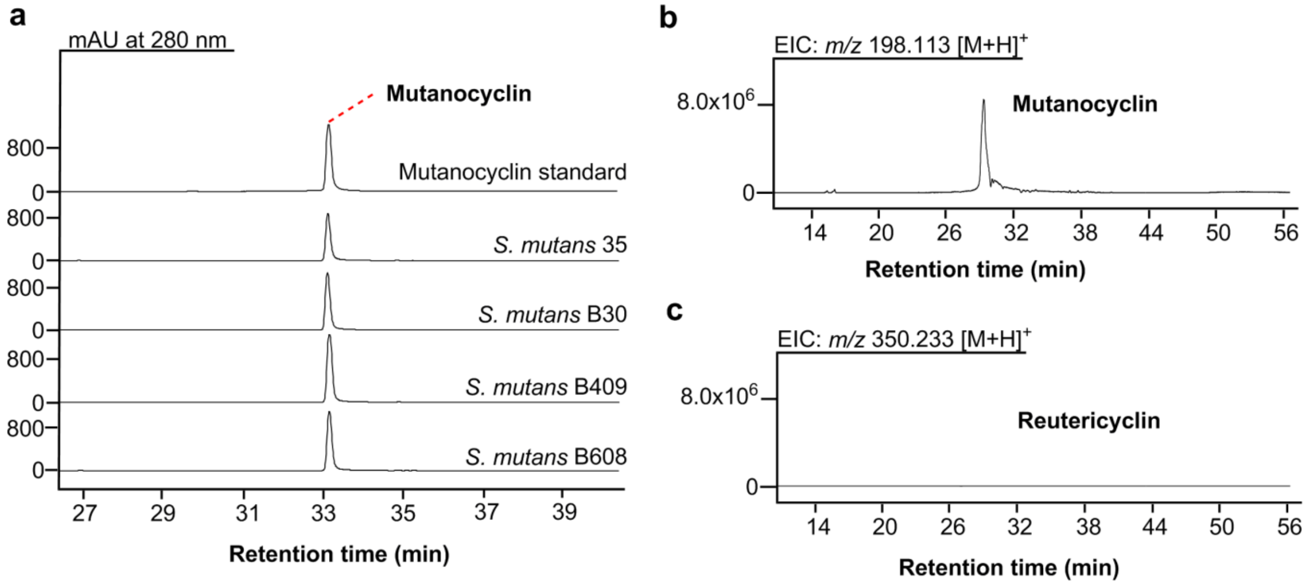

Supplementary Figure 10. HPLC metabolite profiles and HR-LC-MS analyses of the *S. mutans* isolates with BGC1. (a) HPLC traces of the supernatant extracts of the four *S. mutans* isolates with BGC1. HR-LC-MS extracted ion count chromatograms of **(b)** mutanocyclin *m/z* 198.113 $[M+H]^+$ and **(c)** reutericyclin *m/z* 350.233 $[M+H]^+$ in the four *S. mutans* isolates containing BGC1. Only *S. mutans* B35 is shown as representative data. EIC, extracted ion count.

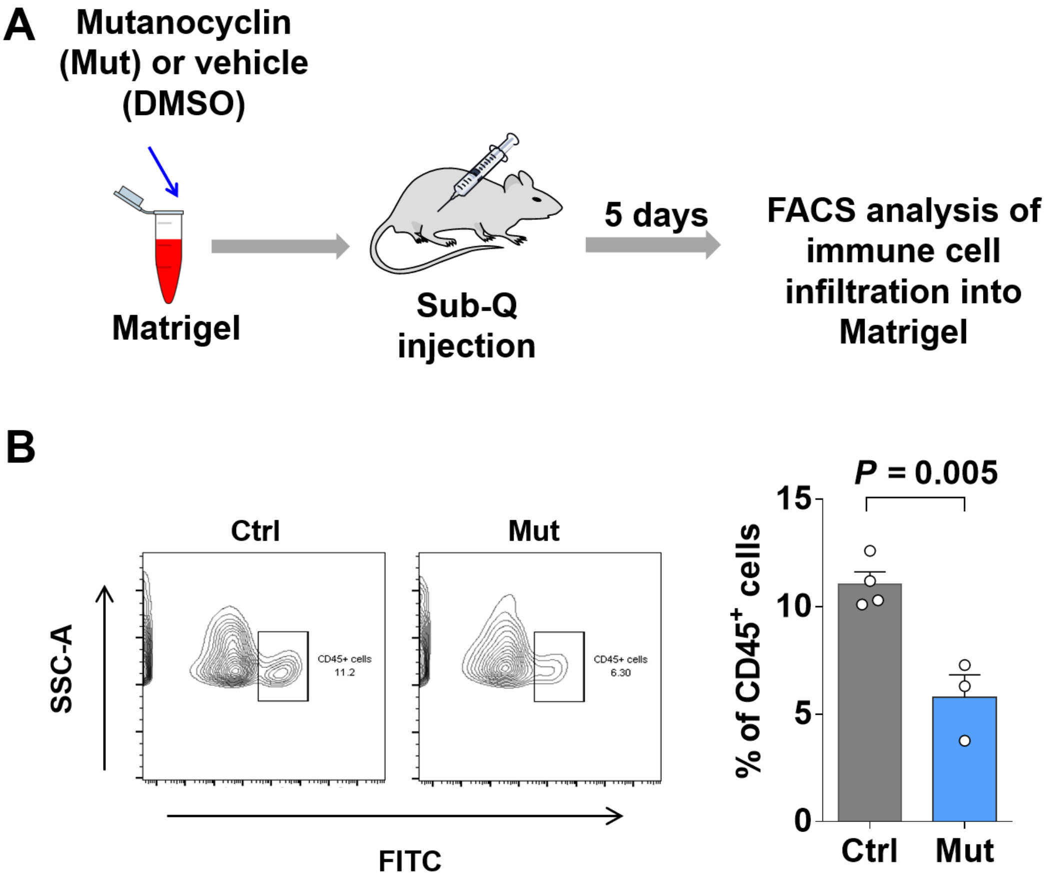

Supplementary Figure 11. Anti-inflammation activity of mutanocyclin. (a) Scheme of the animal experiment: Matrigel containing mutanocyclin or vehicle was subcutaneously injected into the abdominal area of C57BL/6 male mice; after five days, the infiltrated immune cells in the Matrigel plugs were quantified by flow cytometry. **(b)** Quantification of CD45+ immune cells in Matrigel plus. (Left panel) representative flow cytometry images. (Right panel) quantification of $CD45^+$ immune cells in the plug. The results are mean ± SEM, n = 3-4 biologically independent animals, statistical significance was determined using Student's t-test.

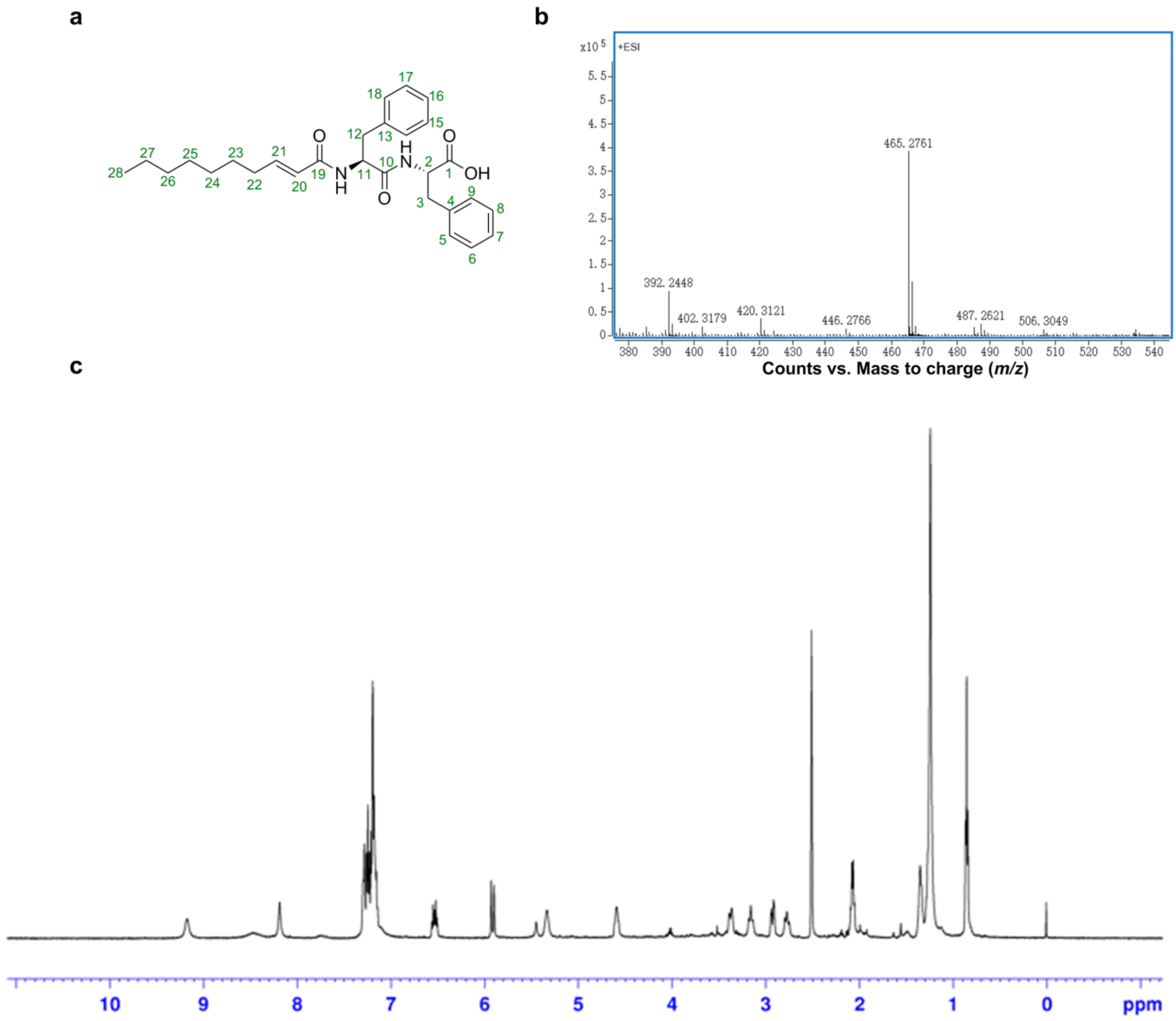

Supplementary Figure 12. Spectral data of SNC1-465. (a) Structure of SNC1-465. **(b)** HR-ESI-MS spectrum of SNC1-465. **(c)** ^{1}H NMR spectrum of SNC1-465 in DMSO-$_{d6}$.

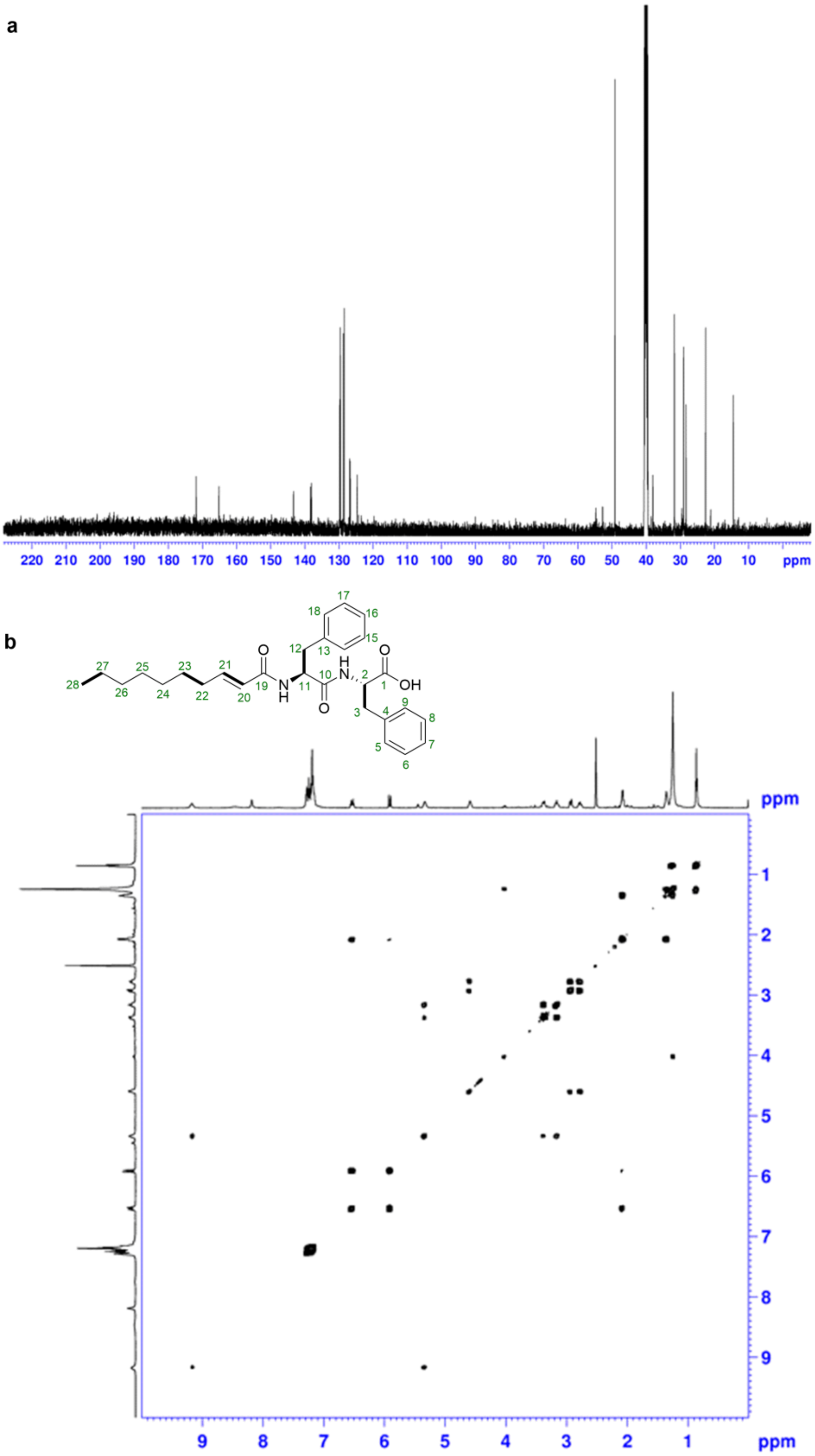

Supplementary Figure 13. Spectral data of SNC1-465. (a) ^{13}C NMR spectrum of SNC1-465 in DMSO-$_{d6}$. **(b)** ^{1}H-^{1}H COSY spectrum of SNC1-465 in DMSO-$_{d6}$. The key COSY correlations are marked on the structure with bold lines.

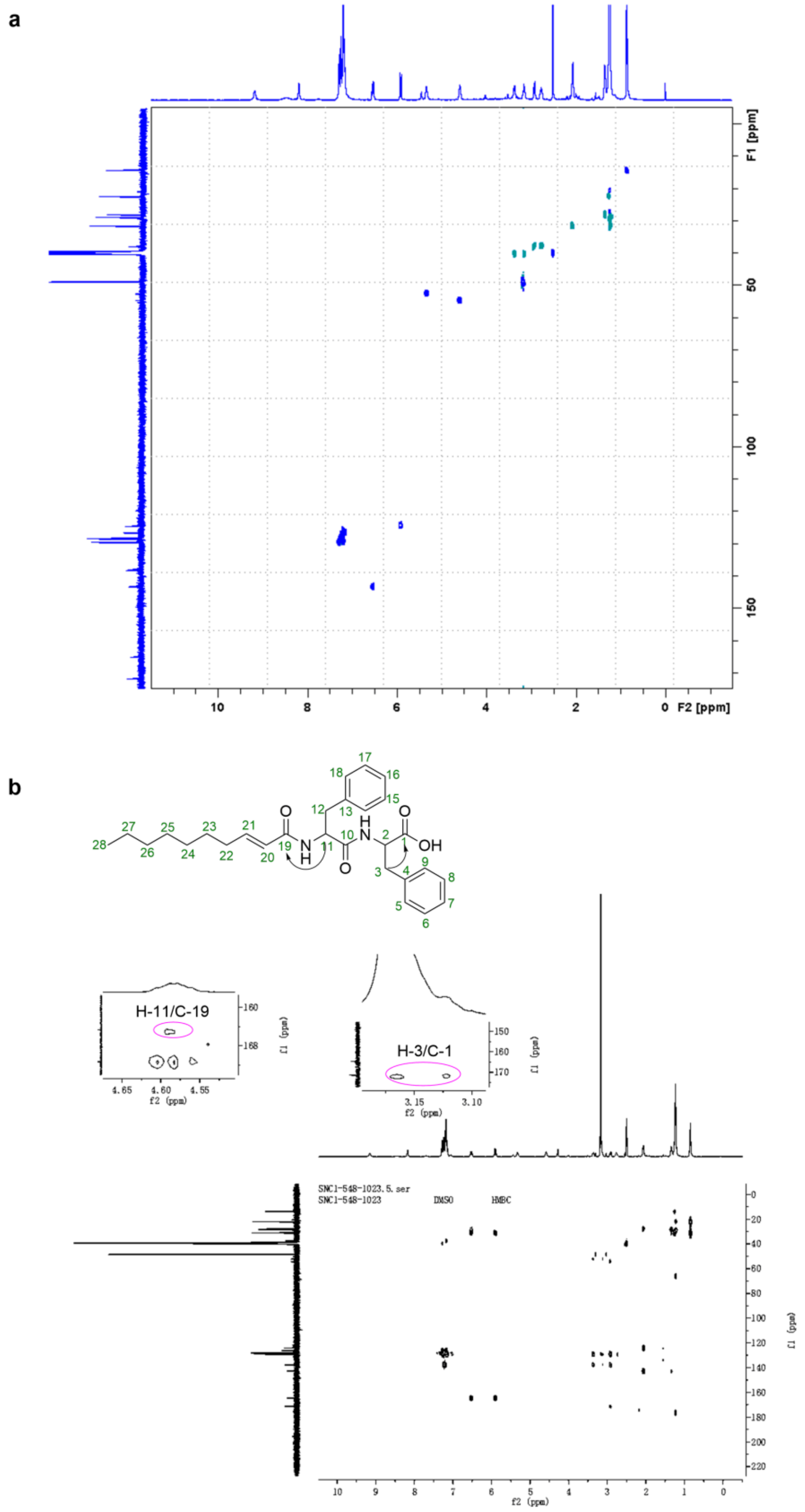

Supplementary Figure 14. Spectral data of SNC1-465. (a) HSQC spectrum of SNC1-465 in DMSO-$_{d6}$. **(b)** HMBC spectrum of SNC1-465 in DMSO-$_{d6}$. The key HMBC correlations are marked on the structure with black arrows and at the spectrum with pink circles.

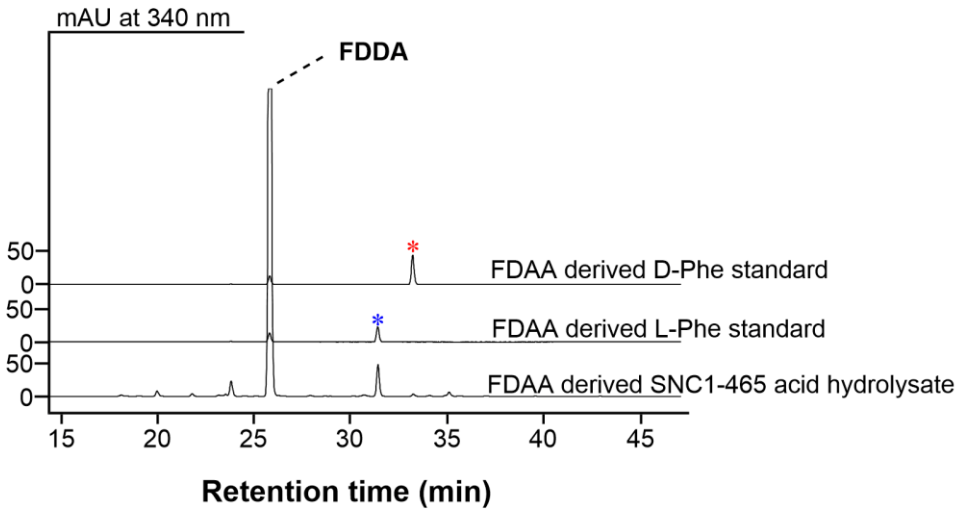

Supplementary Figure 15. HPLC analysis of the FDAA derivatives. FDAA derived *D*-Phe and *L*-Phe are marked with red and blue asterisks respectively. Only *L*-Phe peak was observed in the acid hydrolysate of SNC1-465.

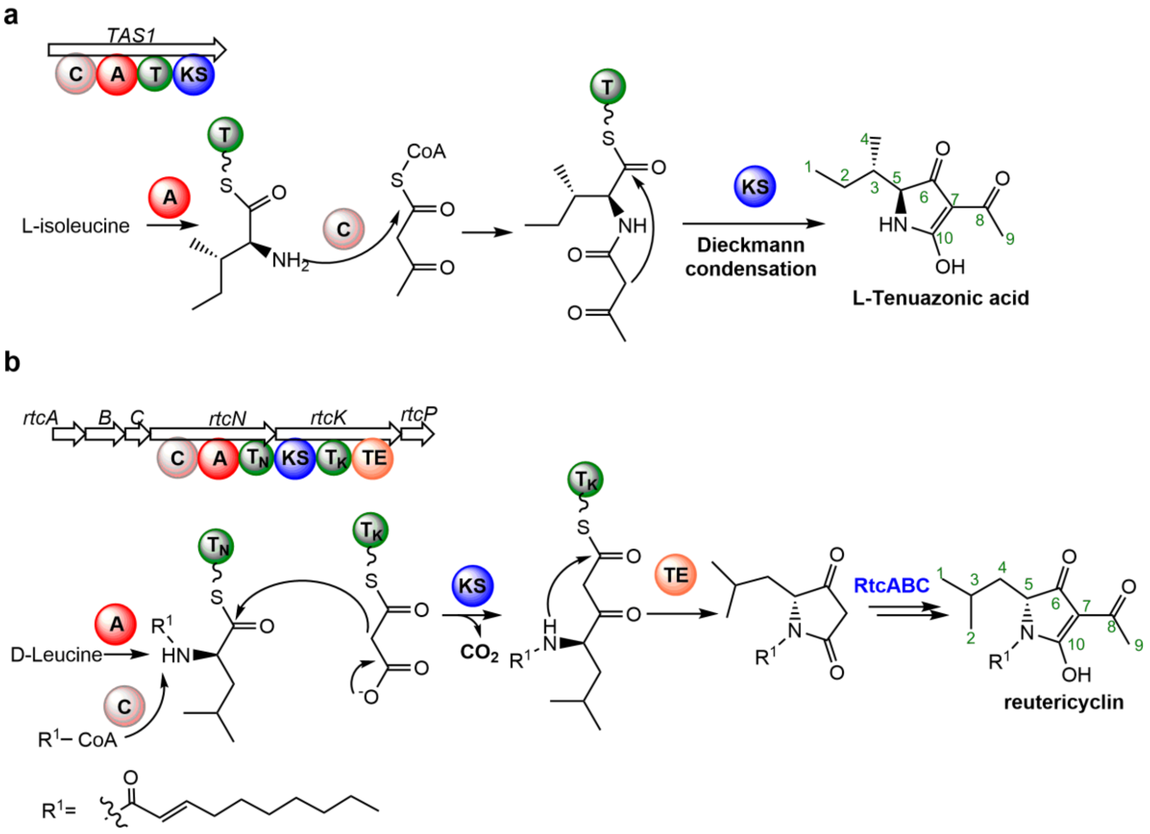

Supplementary Figure 16. The proposed biosynthetic pathways for *L*-tenuazonic acid and reutericyclin. (**a**) The proposed model for *L*-tenuazonic acid biosynthesis. Its C7-acetyl group from acetoacetyl-CoA was incorporated in the first biosynthetic step and the pyrrolidine ring was formed via Dieckmann condensation catalyzed by an atypical KS domain. **(b)** The proposed biosynthetic pathway for reutericyclin. The C7-acetyl group was proposed to be added in the last biosynthetic step and the pyrrolidine ring was suggested to be formed via a TE-catalyzed lactam formation. Functional NRPS and PKS domains are indicated in bold: A, adenylation domain; C, condensation domain; KS, ketosynthase; T, thiolation domain; TE, thioesterase.

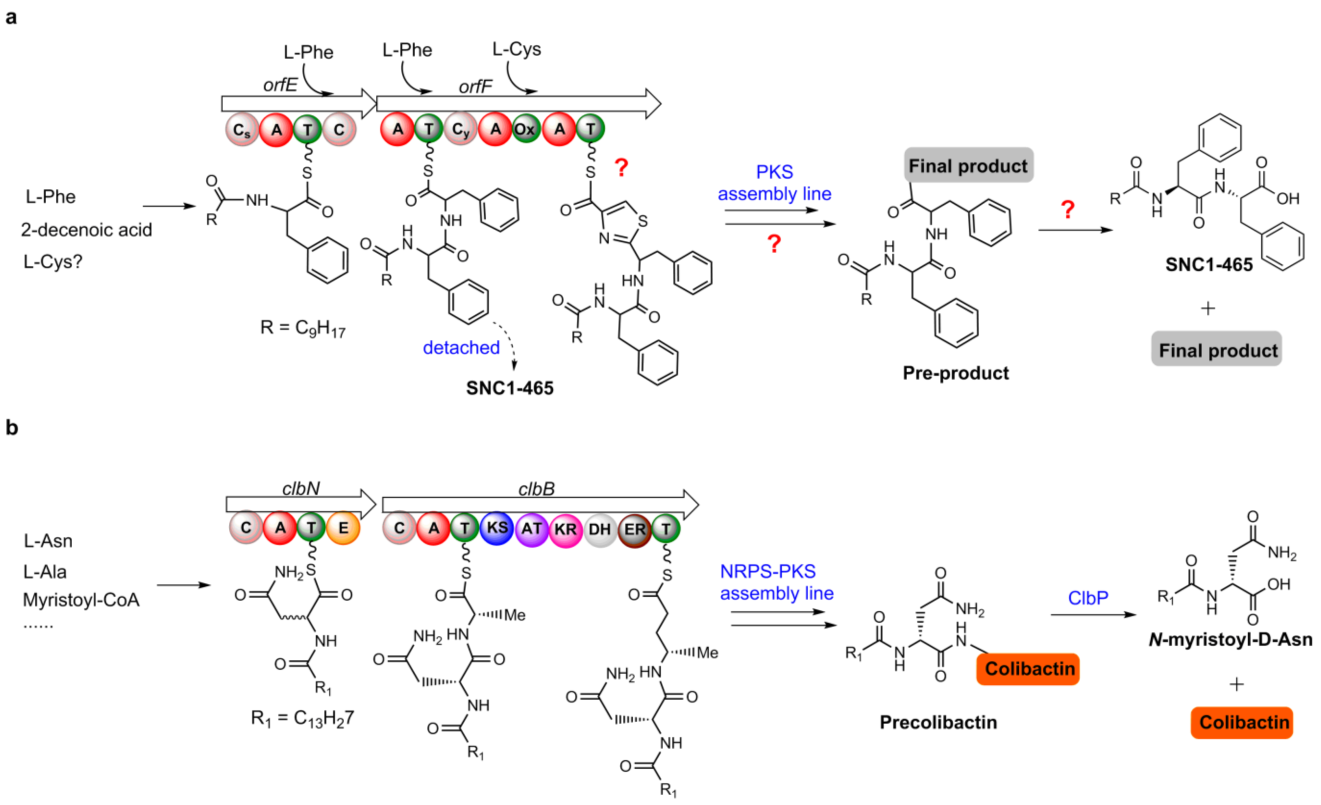

Supplementary Figure 17. The proposed biosynthetic pathways for SNC1-465 and Colibactin. (a) The proposed biosynthetic pathway for SNC1-465. SNC1-465 was proposed to be either a premature product detached from the assembly line or a side product cleaved from the pre-product. **(b)** The proposed biosynthetic pathway for *N*-myristoyl-*D*-Asn and colibactin. Colibactin is firstly synthesized as precolibactin, which is then cleaved by a peptidase ClbP to generate *N*-myristoyl-*D*-Asn and colibactin. Functional NRPS and PKS domains are indicated in bold: A, adenylation domain; C, condensation domain; Cs, starter condensation domain; Cy, heterocyclization domain; Ox, oxidative domain; AT, acyl-transferase; KS, ketosynthase; T, thiolation domain; KR, ketoreductase; DH, dehydratase; ER, enoyl reductase.

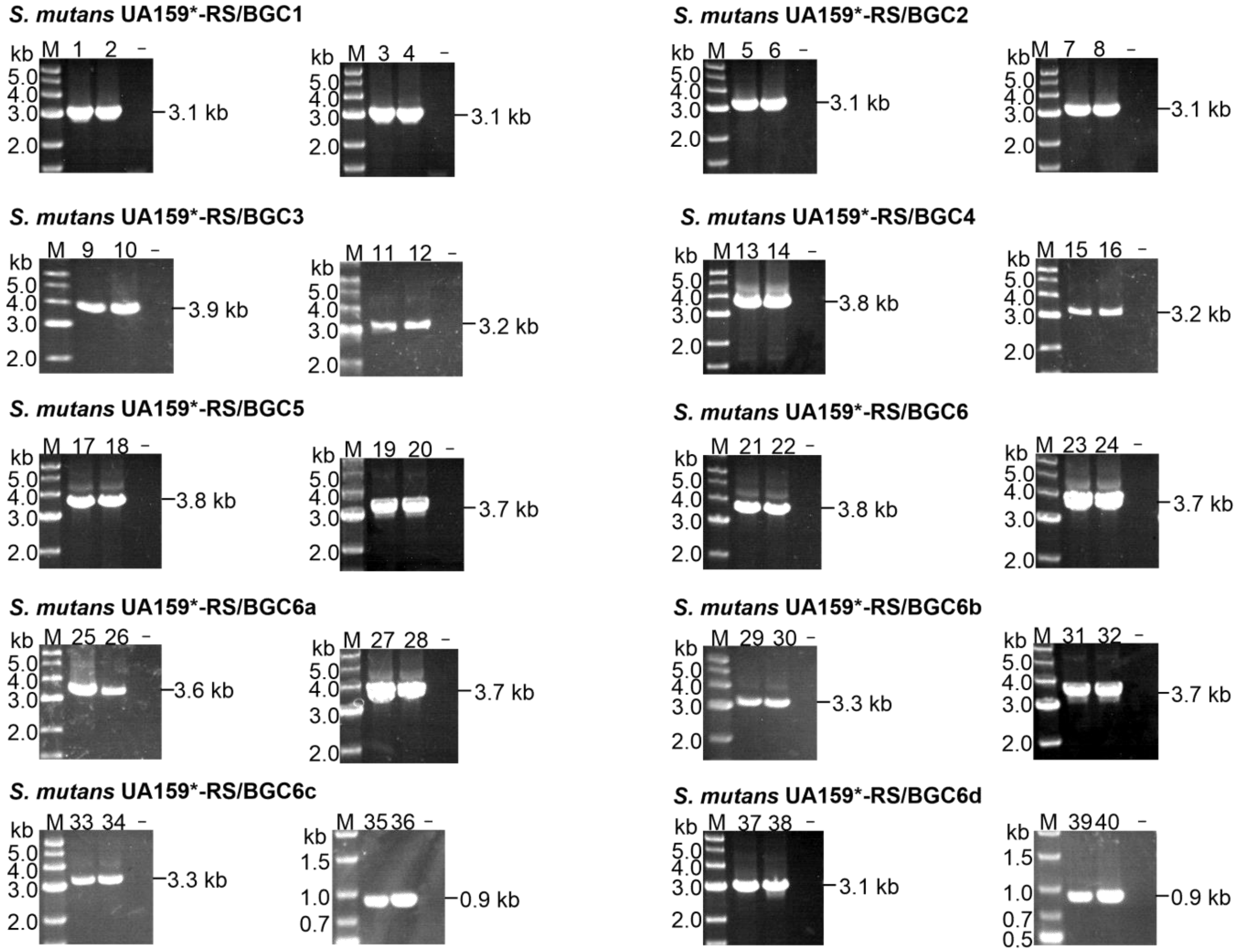

Supplementary Figure 18. PCR verifications of the constructed recipient strains for cloning different BGCs. Lane M, DNA marker; Lane – , negative controls using the genomic DNAs of *S. mutans* UA159* as templates, Lane 1-40, PCR verifications of the correct *S. mutans* UA159*-RS/BGC strains. Source data are provided as a Source Data file.

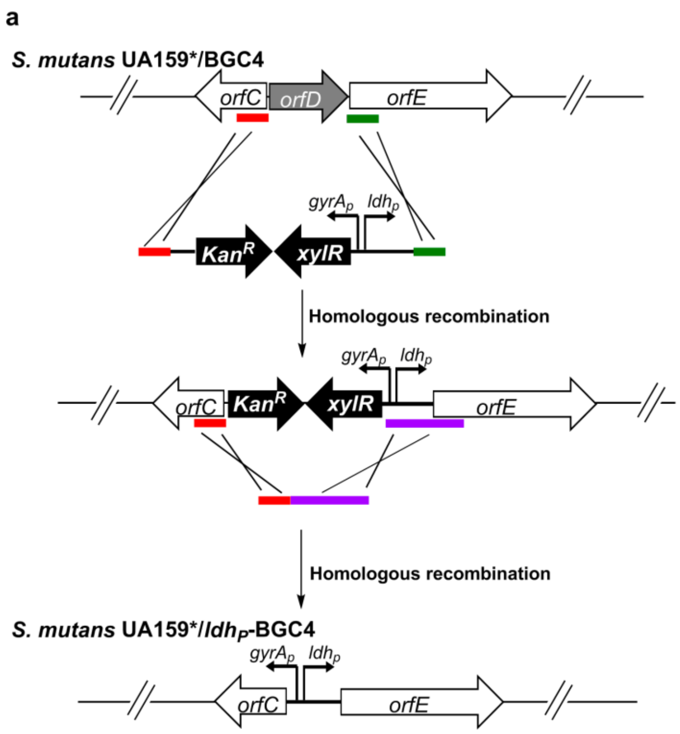

Supplementary Figure 19. Construction of *S. mutans* UA159*/*ldh*$_{P}$-BGC4 and *S. mutans* UA159*/*ldh*$_{P}$-BGC4 Δ*orfF*. (a) The constitutive promoters *gyrA*$_{p}$ and *ldh*$_{P}$ were used to replace the regulatory gene *orfD* in *S. mutans* UA159*/BGC4 to generate *S. mutans* UA159*/*ldh*$_{P}$-BGC4. (**b**) Construction of the *orfF* gene in-frame deletion mutant *S. mutans* UA159*/*ldh*$_{P}$-BGC4 Δ*orfF*. The homologous sequences for recombination are indicated with colorful squares. KRX, KRX cassette as depicted in Fig. 2.

Supplementary Table 1. Background rate comparison of the two counterselection systems

Strains	Selection system	Trials	Number of background cells	Number of total living cells	Average background rate ± s.d.
S. mutans		1	340	23.0×10^{8}	
UA159/BGC1	*pheS**-based	2	380	22.0×10^{8}	$(2.0\pm0.8)\times10^{-7}$
		3	480	16.0×10^{8}	
S. mutans		1	28	30.0×10^{8}	
UA159*/BGC1	*tetM*-based	2	37	35.0×10^{8}	$(1.0\pm0.1)\times10^{-8}$
		3	42	39.0×10^{8}	

Supplementary Table 2. Screening efficiency comparison of the two counterselection systems

Strains	Selection systems	Trials	Number of correct colonies	Number of picked colonies	Average correct rates ± s.d.（%）
S. mutans		1	2	64	
UA159/BGC1	*pheS**-based	2	1	64	2.6±0.9
		3	2	64	
S. mutans		1	15	64	
UA159*/BGC1	*tetM*-based	2	17	64	22.9±3.9
		3	10	64	

Supplementary Table 3. ^{1}H NMR data of the isolated mutanocyclin in $CDCl_3$

NO.	δ_H (ppm, J = Hz)			
	Mutanocyclin (isolated in this study)		(2*R*)-4-Acetyl-1,2-dihydro-5-hydroxy -2-(2-methylpropyl)-3H-pyrrol-3-one	
	A	B	A	B
1	0.99, d(6.0)	0.99, d(6.0)	0.95, d	0.96, d
2	0.99, d(6.0)	0.99, d(6.0)	0.96, d	0.97, d
3	1.77, m	1.77, m	1.73-1.83, m	1.73-1.83, m
4-a	1.46, m	1.46, m	1.43, ddd	1.46, ddd
4-b	1.76, m	1.76, m	1.68-1.73, m	1.67-1.72, m
5	3.87, dd(5.0, 10)	4.03, dd(5.0, 10)	3.85, dd	4.01, dd
6				
7				
8				
9	2.48, s	2.54, s	2.45, s	2.50, s
10				
NH	5.86, s	5.63, s	7.01, s	6.69, s

Note: The isolated mutanocyclin is a mixture of A and B tautomers in $CDCl_3$. The ^{1}H NMR spectrum was measured at 500 MHz. The ^{1}H NMR data of the chemically synthesized mutanocyclin standard in literature[3] are also present comparably.

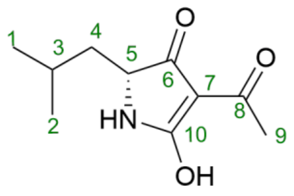

A

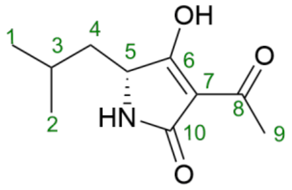

B

Supplementary Table 4. ^{1}**H NMR data of the isolated mutanocyclin in DMSO-*d6***

NO.	δ_H (ppm, J = Hz)
1, 2	0.89, 0.88 (d, J = 1.0 Hz, 6H)
3	1.79 (m, 1H)
4	1.32, 1.48 (m, 2H)
5	3.84 (m, 1H)
6	
7	
8	
9	2.35 (s, 3H)
10	

Note: The ^{1}H NMR spectrum was measured at 500 MHz.

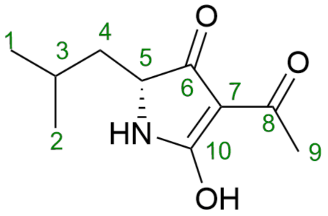

Supplementary Table 5. ^{1}H NMR and ^{13}C NMR data of synthesized mutanocyclin standard in DMSO-*d6*

No.	δ_H (ppm, J = Hz)	δ_C (ppm)
1, 2	0.88, 0.87 (d, J = 1.0 Hz 6H)	21.9, 23.7
3	1.79 (m, 1H)	24.5
4	1.32, 1.48 (m, 2H)	41.2
5	3.86 (m, 1H)	60.8
6		196.0
7		101.1
8		185.2
9	2.35 (s, 3H)	20.0
10		175.4

Note: The ^{1}H NMR and ^{13}C NMR spectra were measured at 500 MHz and 125 MHz respectively.

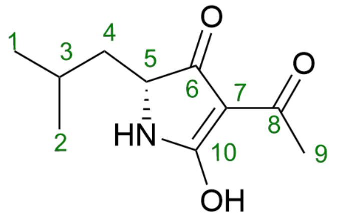

Supplementary Table 6. ^{1}H NMR and ^{13}C NMR data of SNC1-465 in DMSO-$_{d6}$

No.	δ_H (ppm, *J*=Hz)	δ_C (ppm)
1	-	171.9
2	5.33（m, 1H）	52.2
3	3.13, 3.36 (m, 2H)	40.0
4, 13	-	138.1, 138.3
5-9, 14-18	7.14-7.30 (aromatic 10H)	126.7, 126.8, 128.5, 128.6, 129.5, 129.7
10	-	171.4
11	4.59 (m, 1H)	54.2
12	2.77, 2.95 (m, 2H)	37.6
19	-	165.1
20	5.91 (d, 15.4, 1H)	124.6
21	6.53 (m, 1H)	143.3
22	2.08 (m, 2H)	31.7
23	1.35 (m, 2H)	28.3
24-27	1.24 (m, 8H)	22.5, 28.9, 29.0, 31.6
28	0.86 (t, 6.5, 3H)	14.4

Note: The ^{1}H NMR and ^{13}C NMR spectra were measured at 500 MHz and 125 MHz respectively.

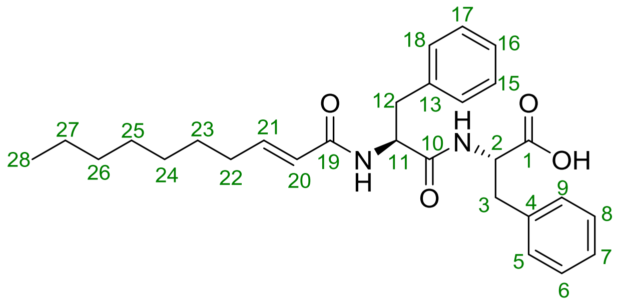

Supplementary Table 7. Antibacterial activity of mutanocyclin

Compounds	MIC/μM							
	E. coli	***B. subtilis***	***S. sanguinis***	***S. oralis***	***S. gordonii***	***L. Lactis***	***V. atypica***	***P. larvae***
**Mut**	>500	>500	>500	>500	500	>500	>500	>500
**Erm**	500	<0.25	<0.25	<0.25	<0.25	<0.25	>500	<0.25
**Tet**	8.0	8.0	0.5	1.0	2.0	0.5	4.0	<0.25

Notes: ***E. coli***, *Escherichia coli* JM109; ***B. subtilis***, *Bacillus subtilis* BS 168; ***S. sanguinis***, *Streptococcus sanguinis* NY101; ***S. oralis***, *Streptococcus oralis* 10557; ***S. gordonii***, *Streptococcus gordonii* 10556; ***L. Lactis***, *Lactococcus Lactis* MG1363; ***V. atypica***, *Veillonella atypica* PK1910; ***P. larvae***, *Paenibacillus larvae* ATCC 13537; **Mut**, mutanocyclin; **Erm**, erythromycin; **Tet**, tetracycline.

Supplementary Table 8. Inhibition activity of SNC1-465 on mushroom tyrosinase

Compound	IC_{50} (μM) [a]
**SNC1-465**	>500
**Kojic acid**	75.9±6.5.

[a]All experiments were performed three times.

Supplementary References

1. Haase, R.G. & Schobert, R. Synthesis of the bioherbicidal fungus metabolite macrocidin A. *Org. Lett.* **18**, 6352-6355 (2016).
2. Schobert, R., Jagusch, C., Melanophy, C. & Mullen, G. Synthesis and reactions of polymer-bound Ph3P=C=C=O: a quick route to tenuazonic acid and other optically pure 5-substituted tetramates. *Org. Biomol. Chem.* **2**, 3524-3529 (2004).
3. Böhme, R., Jung, G. & Breitmaier, E. Synthesis of the antibiotic (R)-reutericyclin via dieckmann condensation. *Helv. Chim. acta* **88**, 2837-2841 (2010).
4. Ajdić, D. et al. Genome sequence of *Streptococcus mutans* UA159, a cariogenic dental pathogen. *Proc. Natl. Acad. Sci. USA* **99**, 14434-14439 (2002).
5. Cornejo, O.E. et al. Evolutionary and population genomics of the cavity causing bacteria *Streptococcus mutans*. *Mol. Biol. Evol.* **30**, 881-893 (2013).
6. Wu, C. et al. Genomic island TnSmu2 of *Streptococcus mutans* harbors a nonribosomal peptide synthetase-polyketide synthase gene cluster responsible for the biosynthesis of pigments involved in oxygen and H_2O_2 tolerance. *Appl. Environ. Microbiol.* **76**, 5815-5826 (2010).
7. Benahmed, F.H. et al. Draft genome sequences of *Streptococcus bovis* strains ATCC 33317 and JB1. *Genome Announc.* **2**, e01012-01014 (2014).
8. Gill, S.R. et al. Insights on evolution of virulence and resistance from the complete genome analysis of an early methicillin-resistant *Staphylococcus aureus* strain and a biofilm-producing methicillin-resistant *Staphylococcus epidermidis* strain. *J. Bacteriol.* **187**, 2426-2438 (2005).
9. Williamson-Charles, H.D. et al. Comparative genomic analyses reveal broad diversity in botulinum-toxin-producing Clostridia. *BMC genomics* **17**, 180 (2016).
10. Jiang, W. et al. Cas9-Assisted Targeting of CHromosome segments CATCH enables one-step targeted cloning of large gene clusters. *Nat. Commun.* **6**, 8101 (2015).
11. Podbielski, A., Spellerberg, B., Woischnik, M., Pohl, B. & Lütticken, R. Novel series of plasmid vectors for gene inactivation and expression analysis in group A streptococci (GAS). *Gene* **177**, 137-147 (1996).
12. Xie, Z., Okinaga, T., Qi, F., Zhang, Z. & Merritt, J. Cloning-independent and counterselectable markerless mutagenesis system in *Streptococcus mutans*. *Appl. Environ. Microbiol.* **77**, 8025-8033 (2011).
13. Xie, Z., Qi, F. & Merritt, J. Development of a tunable wide-range gene induction system useful for the study of streptococcal toxin-antitoxin systems. *Appl. Environ. Microbiol.* **79**, 6375-6384 (2013).
14. Gawron-Burke, C. & Clewell, D.B. Regeneration of insertionally inactivated streptococcal DNA fragments after excision of transposon Tn916 in *Escherichia coli*: strategy for targeting and cloning of genes from gram-positive bacteria. *J. Bacteriol.* **159**, 214-221 (1984).
15. Gutierrez, J.A. et al. Insertional mutagenesis and recovery of interrupted genes of *Streptococcus mutans* by using transposon Tn917: preliminary characterization of mutants displaying acid sensitivity and nutritional requirements. *J. Bacteriol.* **178**, 4166-4175 (1996).
