# Supplementary material for: An anaerobic bacterium host system for heterologous expression of natural product biosynthetic gene clusters
Source: Nat Commun. 2019 Aug 14;10:3665. doi: 10.1038/s41467-019-11673-0 (PMC6694145; doi:10.1038/s41467-019-11673-0)
Supplement: Supplementary file 2 — Reporting Summary [file 41467_2019_11673_MOESM2_ESM.pdf]

nature research

Corresponding author(s): Yihua Chen

Last updated by author(s): Jul 8, 2019

Reporting Summary

Nature Research wishes to improve the reproducibility of the work that we publish. This form provides structure for consistency and transparency in reporting. For further information on Nature Research policies, see Authors & Referees and the Editorial Policy Checklist.

Statistics

For all statistical analyses, confirm that the following items are present in the figure legend, table legend, main text, or Methods section.

n/a	Confirmed	
☐	☒	The exact sample size (*n*) for each experimental group/condition, given as a discrete number and unit of measurement
☐	☒	A statement on whether measurements were taken from distinct samples or whether the same sample was measured repeatedly
☐	☒	The statistical test(s) used AND whether they are one- or two-sided<br>*Only common tests should be described solely by name; describe more complex techniques in the Methods section.*
☒	☐	A description of all covariates tested
☒	☐	A description of any assumptions or corrections, such as tests of normality and adjustment for multiple comparisons
☐	☒	A full description of the statistical parameters including central tendency (e.g. means) or other basic estimates (e.g. regression coefficient) AND variation (e.g. standard deviation) or associated estimates of uncertainty (e.g. confidence intervals)
☐	☒	For null hypothesis testing, the test statistic (e.g. *F*, *t*, *r*) with confidence intervals, effect sizes, degrees of freedom and *P* value noted<br>*Give P values as exact values whenever suitable.*
☒	☐	For Bayesian analysis, information on the choice of priors and Markov chain Monte Carlo settings
☒	☐	For hierarchical and complex designs, identification of the appropriate level for tests and full reporting of outcomes
☐	☒	Estimates of effect sizes (e.g. Cohen's *d*, Pearson's *r*), indicating how they were calculated

Our web collection on statistics for biologists contains articles on many of the points above.

Software and code

Policy information about availability of computer code

Data collection	No software was used for data collection.
Data analysis	The following software was used: Illumina Hiseq 2000, antiSMASH 4.2.0, RBS Calculator v2.0, FlowJo v10, MestReNova v6.1.0, Qualitative Analysis B.07.00, ChemBioDraw Ultra 14.0.

For manuscripts utilizing custom algorithms or software that are central to the research but not yet described in published literature, software must be made available to editors/reviewers. We strongly encourage code deposition in a community repository (e.g. GitHub). See the Nature Research guidelines for submitting code & software for further information.

Data

Policy information about availability of data

All manuscripts must include a data availability statement. This statement should provide the following information, where applicable:

- Accession codes, unique identifiers, or web links for publicly available datasets
- A list of figures that have associated raw data
- A description of any restrictions on data availability

The BGCs cloned in this study have been deposited into GenBank under the accession numbers: MK144293 (BGC1), NC_002976.3 (BGC2), NZ_AUZG00000000.1 (BGC3), AHRZ00000000.1 (BGC4), NZ_LFPM00000000.1 (BGC5), and MK144294 (BGC6). S. mutans 35 genome sequence has been deposited into GenBank with the accession number: SZVN00000000.

Field-specific reporting

Please select the one below that is the best fit for your research. If you are not sure, read the appropriate sections before making your selection.

☒ Life sciences ☐ Behavioural & social sciences ☐ Ecological, evolutionary & environmental sciences

For a reference copy of the document with all sections, see nature.com/documents/nr-reporting-summary-flat.pdf

Life sciences study design

All studies must disclose on these points even when the disclosure is negative.

Sample size	No particular methods were used to determine sample size. The background rate comparison and screening efficiency comparison of the two counterselection systems were generally determined based on at least three independent experiments. The NabLC technique was used to clone six different BGCs from diverse anaerobic bacteria including Streptococcus, Staphylococcus, and Clostridium.
Data exclusions	No data exclusions.
Replication	All attempts at replication were successful.
Randomization	For cloning BGCs with the NabLC technique in this study, positive colonies were randomly picked for further screening.
Blinding	Blinding was not relevant to our study as there were no differing selection or exclusion criteria between sampled participants.

Reporting for specific materials, systems and methods

We require information from authors about some types of materials, experimental systems and methods used in many studies. Here, indicate whether each material, system or method listed is relevant to your study. If you are not sure if a list item applies to your research, read the appropriate section before selecting a response.

Materials & experimental systems

n/a	Involved in the study	
☐	☒	Antibodies
☒	☐	Eukaryotic cell lines
☒	☐	Palaeontology
☐	☒	Animals and other organisms
☒	☐	Human research participants
☒	☐	Clinical data

Methods

n/a	Involved in the study	
☒	☐	ChIP-seq
☐	☒	Flow cytometry
☒	☐	MRI-based neuroimaging

Antibodies

Antibodies used	Purified anti-mouse CD45 Antibody (catalog number: 103107; clone: 30-F11, dilution ratio: 1:500)
Validation	Purified anti-mouse CD45 Antibody: Validation profile is on the manufacturer's website (https://www.biolegend.com/en-us/products/purified-anti-mouse-cd45-antibody-102).

Animals and other organisms

Policy information about studies involving animals; ARRIVE guidelines recommended for reporting animal research

Laboratory animals	6-week-old C57BL/6 male mice was used in the study.
Wild animals	The study did not involve wild animals.
Field-collected samples	The study did not involve samples collected from the field.
Ethics oversight	Animal experiments were conducted in accordance with the protocols approved by the Institutional Animal Care and Use Committee (IACUC) of University of Massachusetts Amherst.

Note that full information on the approval of the study protocol must also be provided in the manuscript.

Flow Cytometry

Plots

Confirm that:

☒ The axis labels state the marker and fluorochrome used (e.g. CD4-FITC).

☒ The axis scales are clearly visible. Include numbers along axes only for bottom left plot of group (a 'group' is an analysis of identical markers).

☒ All plots are contour plots with outliers or pseudocolor plots.

☒ A numerical value for number of cells or percentage (with statistics) is provided.

Methodology

Sample preparation	Briefly, 0.25 mL growth factor-reduced Matrigel (BD Biosciences, San Jose, CA), which was pre-mixed with mutanocyclin or DMSO vehicle, was subcutaneously injected into 6-week-old C57BL/6 male mice in the abdominal area. After 5 days, the mice were euthanized to dissect the implanted Matrigel plugs. The plugs were digested using Corning® cell recovery solution (Corning, NY), filtered through 70 µm cell sorters (BD Biosciences, San Jose, CA) to obtain single cell suspension, which were stained with FITC-conjugated anti-mouse CD45 antibody and isotype control antibody (BioLegend, San Diego, CA).
Instrument	The stained cells were analyzed using BD LSRFortessa™ cell analyzer (BD Biosciences, San Jose, CA).
Software	Flow cytometry plots were analyzed on FlowJo v10.
Cell population abundance	The purity of sorted sample was not determined post-sort.
Gating strategy	Gating and cell identification strategies are as follows: cell doublets and clumps were eliminated using FSC-H vs. FSC-A gating, and debris was eliminated using FSC-A vs. SSC-A. Dead cells were gated out using Zombie Violet™ dye.

☐ Tick this box to confirm that a figure exemplifying the gating strategy is provided in the Supplementary Information.
