# Supplementary material for: An anaerobic bacterium host system for heterologous expression of natural product biosynthetic gene clusters
Source: Nat Commun. 2019 Aug 14;10:3665. doi: 10.1038/s41467-019-11673-0 (PMC6694145; doi:10.1038/s41467-019-11673-0)
Supplement: Supplementary file 3 — Description of Additional Supplementary Files [file 41467_2019_11673_MOESM3_ESM.docx]

**Description of Additional Supplementary Files**

File Name: Supplementary Data 1
Description: Bacterial strains and plasmids used in this study

File Name: Supplementary Data 2
Description: Primers used in this study
